# Recent Advances in MEMS Actuators for Microfluidic Applications: Emerging Designs, Multiphysics Modeling, and Performance Optimization

**DOI:** 10.3390/mi17030347

**Published:** 2026-03-12

**Authors:** Oliur Rahman, Md Mahbubur Rahman, Onu Akter, Md Nizam Uddin, Md Shohanur Rahman, Sourav Roy, Md Shamim Sarker

**Affiliations:** 1 Department of Mechanical Engineering, Khulna University of Engineering & Technology, Khulna 9203, Bangladesh; rahman2005029@stud.kuet.ac.bd; 2 Department of Textile Engineering, Khulna University of Engineering & Technology, Khulna 9203, Bangladesh; akter1921011@stud.kuet.ac.bd; 3 James C. Morriss Division of Engineering, Texas A & M University-Texarkana, 7101 University Ave., Texarkana, TX 75503, USA; 4 Department of Mechatronics Engineering, Khulna University of Engineering & Technology, Khulna 9203, Bangladesh; shohan@mte.kuet.ac.bd (M.S.R.); s.roy@mte.kuet.ac.bd (S.R.); 5 Department of Bioengineering, Graduate School of Engineering, The University of Tokyo, 7-3-1 Hongo, Bunkyo-ku, Tokyo 113-8656, Japan; sarker@bioxide.t.u-tokyo.ac.jp

**Keywords:** MEMS actuators, microfluidics, lab-on-a-chip, multiphysics modeling, performance optimization, AI-assisted design, microfabrication

## Abstract

This review deals with the development and progress of micro-electromechanical systems (MEMS) actuators, which are needed in microfluidic applications, such as lab-on-a-chip and diagnostics. In the last 10 years, there have been tremendous advances in materials, microfabrication and computational modeling that have increased the functionality and scope of MEMS-based microfluidic actuation. This study classifies MEMS actuators on the basis of the physical method of actuation, including electrostatic, piezoelectric, and pneumatic actuation designs, in comparison with their application in pumping, valving, and droplet control. It examines the suitability of emerging structural and functional materials, such as piezoelectric thin-films and electroactive polymers, paying special attention to their reliability and biocompatibility. It also highlights the progress in multiphysics modeling that incorporates electrical, thermal, mechanical, and fluidic models, which facilitates the efficient design and performance optimization procedures. Other trends are multifunctional actuators with built-in sensing capability and the use of artificial intelligence (AI)-assisted design in production. With these developments, however, there exist issues of power efficiency, thermal control, fabrication uniformity and operational durability, and also the absence of standardized benchmarking. Finally, future research directions are outlined, including hybrid MEMS actuation, intelligent microfluidic operations, to improve the performance of the system and enable the transfer of the lab demonstrations to the large scale application of the system.

## 1. Introduction

Micro-electromechanical systems (MEMS) actuators are small transducers which convert electrical, thermal, magnetic or other types of energy into precise mechanical motion or force [1]. Made by photolithography, these transducers are the basic muscle of microsystems, allowing for dynamic physical control at micrometer and millimeter scales [2]. MEMS actuators are significant because they offer actuation with high control and rapid and localized actuation that is essential in the operation of modern technologies. They can be used in an extensive variety of applications, such as in stabilizing smartphone cameras with voice-coil motors [3], in deploying airbags in cars with inertial sensors [4], steering optical beams in telecommunications [5] and targeted drug delivery in implantable medical devices [6]. Of particular importance are the current developments in MEMS actuators in microfluidic applications, which aim for a new design, advanced multiphysics models, and strict performance optimization. These advances are driving the limits of Lab-on-a-Chip (LoC) systems, which is capable of controlling fluids and particles on the nanoliter scale with precision that has never been realized before. This development is essential for facilitating next-generation point-of-care diagnostics, high-throughput single-cell analysis, and portable environmental monitoring systems, and advanced chemical and biological analysis is more accessible, efficient and powerful than ever before [7,8].

MEMS has experienced a geometric increase in its application within the last two decades, with microfluidic applications emerging as a strong and transformative field of study. This onslaught has of course been associated with an increase in the number of review articles that have tried to depict the state of the art. Various review articles have provided comprehensive descriptions of the larger MEMS and microfluidics environment. The work by Senturia [9] set the fundamental guidelines of a microsystem design, whereas the work of Madou [2] described the full arsenal of fabrication methods at the disposal of MEMS engineers. Squires and Quake [10] described the physics and possibilities of microfluidics in a very eloquent way, and the area of MEMS actuators has been brought to a point of growth. Many reviews have also been dedicated to MEMS actuators. Actuation principles were explained in an early general overview by Judy [11] and their performance measure was compared by Bell et al. [12]. However, the older literature preempts many of the recent advances in materials, methods of fabrication, and multiphysics modeling that are important in the MEMS actuator design today. Judy [11] has outlined a historical and broad study of MEMS fabrication, design principles and applications, and in any case, does not reflect the challenges of fluid–structure interaction that exist in contemporary microfluidic systems.

In the microfluidics space, most reviews have been of a component-based nature. Laser and Santiago [13] introduced a breakthrough review on micropumps, which lacked a discussion of the new actuation schemes such as electrochemical or more advanced magnetohydrodynamic (MHD) principles, and its discussion of how to optimize performance had been restricted to simple geometrical parameters. Woias [14] reported a brief description of micropumps, though did not give critical evaluations of non-mechanical (e.g., electrokinetic, electrowetting) pumps and their ability to operate with biological fluids. Nguyen and Wu [15] have offered a thorough and comprehensive review of the micromixers, including passive and active mixing strategies, the actuation mechanism of the mixers, and their fabrication methods. However, it predates much of the more recent developments in the system integration of microfluidics, such as the introduction of concepts of soft and adaptive actuation, data-driven and artificial intelligence-assisted design paradigms, and the reliability and long-term performance studies needed to translate these concepts into robust lab-on-a-chip and biomedical systems. Similarly to the mechanical micropumps reviewed by Amirouche et al. [16], a significant gap exists in the progress made in electrokinetic, acoustic, and surface-tension-based actuation modalities that are essential to lab-on-a-chip system development. On the subject of microneedles, other important reviews have been made by researchers. Kim et al. [17], have presented a detailed review of the microneedles in drug and vaccine delivery, including the types of needles, fabrication methods and biomedical usage. Their work, however, mainly covers performance in terms of delivery and clinical relevance, but not much discussion of mechanical failure mechanisms, detailed fluidic transport in hollow microneedles or the obstacles to integration with other active microfluidic devices like micropumps. Donnelly et al. [18] provided a comprehensive review of hydrogel-forming microneedles but only did so in relation to their particular category, not the entire range of solid, coated and dissolvable microneedles and their required actuation and integration requirements.

Although a review by Sachdeva and Banga [19] was extensive regarding the applications of therapeutic use of the devices and regulatory concerns, it was not as comprehensive about the multiphysics of needle insertion, the optimization of painless insertion, or the closed-loop control systems needed to achieve smart deliveries of responsive drugs.

Beyond pumps and needles, there are broader reviews on Bio-MEMS, but they lacked detail on the actuation needed. Grayson et al. [20] have also discussed a short discussion of different integrated MEMS devices but failed to give a single unified presentation as to the design, modeling, fabrication and performance optimization of the actuators that drive them. Prausnitz and Langer [21] analyzed transdermal drug delivery systems, detailing advanced actuation methods such as microneedles and active permeation enhancers, and critically analyzed their working principles and performance limitations under realistic biological conditions, including challenges in dosing precision, skin variability, and long-term biocompatibility.

This review provides a critical assessment of the progress made in MEMS actuators to be used in microfluidic applications, focusing on the links between design concepts and multiphysics simulations, and maximizing the performance of the actuators. It discusses the essential actuation processes, electrostatic, electrothermal, piezoelectric, electromagnetic and surface-tension, and their applications in pumps, valves, mixers and droplet manipulators. In contrast to the earlier literature that conducts studies on individual aspects, this review provides a more comprehensive perspective at the system level, and it deals with the combination of sophisticated simulation and material science with smart control. It highlights the need to focus on predictive design with high-fidelity modeling, geometry optimization based on AI and the significance of reliability to enable clinical integration. This review can be a key reference for scientists and researchers working in the emerging area of bio-MEMS and lab-on-a-chip technology.

## 2. Classification of MEMS Actuators for Microfluidics

**Figure 1:**
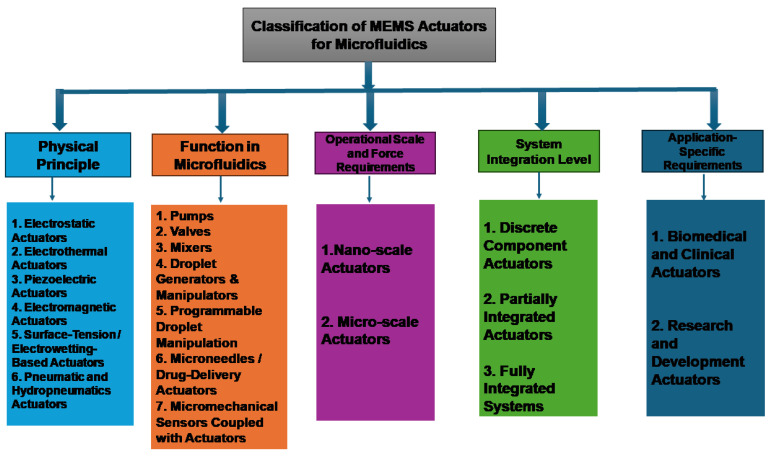
Major classification of MEMS actuator for microfluidics.

The great variety of microfluidic applications requires an equally large variety of actuation solutions. This part is a systematic classification of MEMS actuators in several respects, based on their basic physical principle, their functional application in microfluidic systems, the scale of their operation, and their level of integration into systems [10]. Figure 1 provides a schematic categorization of MEMS actuators to be used in microfluidic applications in terms of physical actuation principles, functional role, size of operation, system integration degree, and application requirements.

### 2.1. By Physical Principle

#### 2.1.1. Electrostatic Actuators

The electrostatic actuators find many applications in MEMS because of their high dynamic response, low static power consumption and compatibility with standard microfabrication processes. In microfluidic systems, the most common architecture for achieving electrostatic actuation is in parallel plate or comb drive systems, which are developed for microscale systems, and should be clearly differentiated from macroscopic systems using electrostatic actuation.

##### Governance of Relation and Working Principle

Electrostatic actuation is generally treated by an energy-based formulation. For a voltage-driven electrostatic actuator, the electrostatic force is found by differentiating the stored electrostatic energy with bucking direction. For a traditional parallel plate type of actuator, the electrostatic force can be defined as [22,23].

$$F=\frac{1}{2}\varepsilon \frac{W\;L}{g^{2}}V^{2}$$

where ε = ε_0_ε_r_ is the permittivity of the dielectric medium between the electrodes, *W* and *L* are the effective electrode width and length, respectively, *V* is the applied voltage, and *g* is the instantaneous electrode gap. This expression is derived assuming constant-voltage actuation, negligible fringing fields, and uniform electric field distribution.

##### Pull-In Instability and Architecture Implications

One of the basic limitations of parallel plate electrostatic actuators is static pull in instability. Under the classical model, which regards the piezoelectric effect as linear spring movement, there is a stable equilibrium only up to about one-third of the initial gap between the electrodes. Beyond this critical displacement, the electrostatic force becomes a quicker force than the mechanical restoring force, so the movable electrode collapses suddenly [24]. To counter the effect of gap closure pulling-in, comb-drive actuators are typically used. In comb-drive architecture, electrostatic force is created by the variation in lateral capacitance, in contrast to vertical gap reduction, which allows for larger stable displacements (in the desired actuation direction) and is attractive for microfluidic MEMS applications, which require repetitive and reliable operation [25].

##### Integral Limitations of Micro Fluidic

When electrostatic actuators are used in close proximity to or in a liquid environment, there are other limitations. Electrodes have to be electrically isolated and passivated to prevent leakages and electrolysis, as well as corrosion. The reliability of the devices is frequently controlled by the charging of the dielectric, dielectric breakdown, or the charging limits of the dielectric, as well as the stiction effect of the dielectrics or wear of contacts per repeated actuation cycle. These effects are especially pronounced for aqueous or conductive fluids and place stringent requirements on the materials, packaging and driving schemes [26,27].

##### Practical Design Trade-Offs

Electrostatic force is proportional to the square of potential and inversely proportional to the square of gap between the electrodes, which means that reducing the gap will be a viable adaptation to increase the actuation force at moderate voltages [22]. However, high reduction in the electrode gap causes increasing susceptibility to pull-in instability, dielectric breakdown and fabrication variability, which limits the operating window of the parallel plate electrostatic actuators. In microfluidic environments, these risks are increased further with the presence of liquids, which add another set of challenges in terms of dielectric charging, leakage currents, stiction and packaging reliability. Consequently, although electrostatic actuation shows excellent performances for obtaining fast and low-power microfluidic actuation, the realization of the overall system needed the secular co-design of mechanical structures, dielectric layer and system-level packaging for stable and reliable operation [28].

#### 2.1.2. Electrothermal Actuators

Electrothermal actuators work on the principle of thermal expansion of structural materials by Joule heating. The basic governing relationship is that of thermal strain.

*ε* = *αΔT*

where ε is the mechanical strain, α is the coefficient of thermal expansion (CTE) of the actuator material, and ΔT is the temperature rise, relative to the ambient [29].

Thus, the corresponding actuator displacement is not only a material property, and strongly depends on the device geometry, as well as structural constraints, considerations of thermal boundary conditions and heat sinking and thermal isolation features. To be clear about this, the symbol ε is used here exclusively to refer to strain and should not be confused with the permittivity that is used in electrostatic actuation models elsewhere [30]. Common configurations of electrothermal actuator types are chevron (V) shaped, providing in-plane motion; bimorph types provide out-of-plane bending (via differential thermal expansion of gammat materials); and hot–cold arm or U-shaped types cue the asymmetric heating to provide rotary or translational motion. Chevron actuators are commonly employed because of their small footprint, mechanical strength and relatively high force output [31]. The force of electrothermal MEMS actuators usually produces tens of micronewtons to a few millinewtons, depending on geometry, material stack and operating temperature increase [32]. The intrinsic displacement of electrothermal actuators typically falls in the sub-micrometer to tens of micrometers range, whereas effective strokes in the tens and hundreds of micrometers range (almost in the sub-millimeter scale) are realized only by compliant or flexural amplification mechanisms. Displacements on the order of millimeters or centimeters are not representative of monolithic electrothermal actuators and are inconsistent with the actuation mechanism of MEMS electrothermal actuators [33]. One of the definitions of electrothermal actuation is the thermally limited response time. The dynamic behavior is controlled by the device thermal time constant, which is a function of the thermal mass of the device actuator and the effectiveness of the dissipation of heat to the substrate and surroundings. Consequently, electrothermal actuators usually have response times in the millisecond to second range, which is slower than electrostatic actuation, but adequate for many of the operations in microfluidics, such as pumping, valving, and positioning [34].

##### Thermal Issues in Integrating Microfluidics

When electrothermal actuators are integrated with microfluidic systems, heat produced inside the actuator is capable of heat conduction to neighboring fluidic channels. This can lead to a localized fluid temperature increase, thermal drift and degradation of temperature-sensitive biological samples. In the case of polymer-based microfluidic platforms (e.g., PDMS), this is not only because of the repeated thermal cycling, which can also cause material softening and dimensional drift, and long-term reliability issues. These effects are of particular importance in biological and biochemical applications where cell viability, protein stability and reaction kinetics are found to be temperature dependent [35].

##### Design and Mitigation Strategies

To limit undesired thermal coupling while maintaining actuation performance, several mitigation strategies are commonly used: (i) thermal isolation structures, e.g., narrow beams or air gaps to localize the heating effect, (ii) pulsing or operating at low duty cycle in order to limit the steady-state temperature rise, (iii) improved heat sinking to a substrate or using dedicated thermal paths and (iv) physical separation of hot actuator elements from fluidic channels with membranes or layers, based on low thermal conductivity [36]. With suitable thermal management, electrothermal actuators are still interesting for microfluidic systems in which high force output, low driving voltage, and integrated small size are required, while a relatively high power consumption is accepted, generally in the 10–100 mW range [31,37].

**Figure 2:**
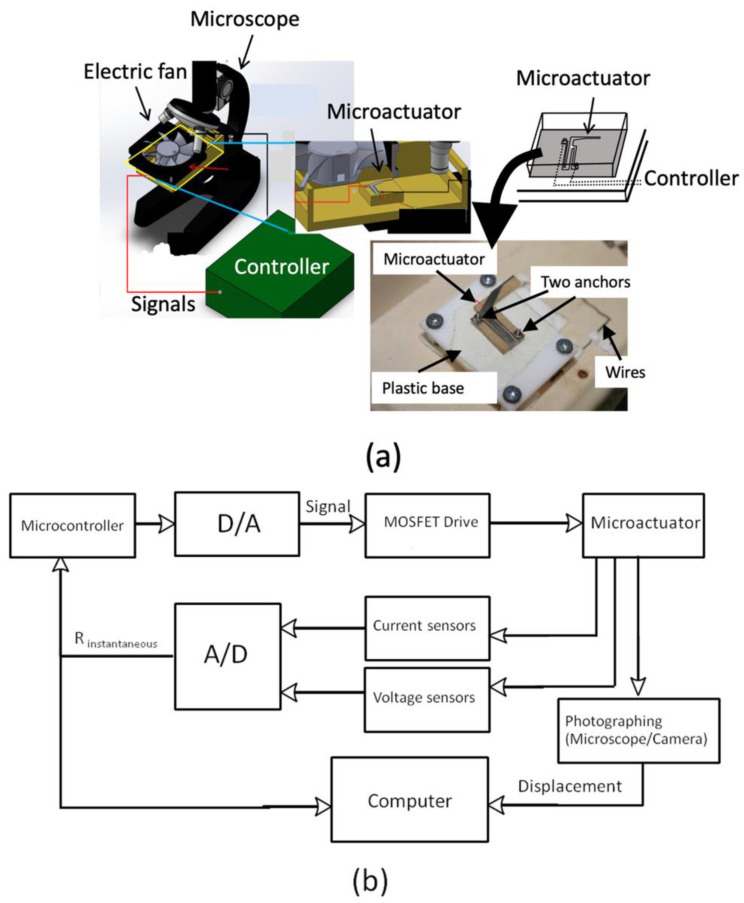
Procedural experimental design to describe electrothermal. An example of MEMS actuators (**a**) observation of optical displacement under controlled thermal conditions and (**b**) driving and sensing circuitry of electricity. D/A and A/D represent digital-to-analog and analog-to-digital converters, respectively [38].

A representative experimental setup, represented in Figure 2, demonstrates typical characterization and control practices when using electrothermal MEMS actuators, as opposed to introducing a new experimental methodology. It also demonstrates an experiment conducted on the mechanical and electrical behavior of the microactuator. To avoid the disturbances that are not tightly constrained, the microactuator was placed in a partially closed box that had a glass cover. The anchors of the microactuator were installed on the plastic supports that were installed inside the box. The box was then mounted on the microscope stage to observe the motion of the microactuator simultaneously through the glass cover, as was observed in Figure 2a. An electric fan was also set up over the set-up to make flow disturbances where necessary. It was also utilized to cool down the temperature of circuit boards and all the electronic devices. Ohm law was used to test the electrical resistance of the actuators by observing the instantaneous potential voltage and current of the heating/sensing elements. The microactuator was actuated and monitored by connecting the experimental circuit, as in Figure 2b. The current circuit control system was used to pass the electrical current through the microactuator structure. This control system comprised the power supply, two hall-effect current sensors, and a MOSFET Vishay IRL510 (Vishay Intertechnology, Inc., Malvern, PA, USA) (controlled by a signal of the Arduino MEGA 2560), with the help of a 12-bit digital-to-analog converter MCP 4725 (Microchip, Tampa, FL, USA).

This experimental design creates salient issues of actuation with electrothermal methods, which include thermal drift, changes in resistance during actuation, and the necessity of electrical and mechanical concomitancy in microfluidic systems.

#### 2.1.3. Piezoelectric Actuators

Piezoelectric actuators work on the inverse piezoelectric effect, the phenomenon that an applied electrical field causes electromechanical de-endowment in a piezoelectric material. The constitutive relation for this effect may be expressed as

*S* = *dE*

where S denotes the induced mechanical strain, d is the piezoelectric strain coefficient, and E is the applied electric field [39].

The coefficient that a particular axis convention and actuation mode is dependent on the axis convention and actuation mode: $d_{33}$ is the amount of strain that is produced along the direction of the electric field when the electric field is applied parallel to that direction, and $d_{31}$ is the amount of strain that is produced perpendicular to the direction of the applied electric field. When actuator displacement (stroke) is mentioned, it is a result of the product of this strain and device geometry, which can be enhanced again by compliant or mechanically enhanced structures [40].

##### Material Platforms: Thin Film Piezoelectric MEMS vs. Bulk Piezo Actuators

To achieve clarity and quantitative consistency, it is necessary to differentiate between bulk- or stack-type piezoelectric actuators and thin-film piezoelectric MEMS actuators, since these types of actuators have significantly different material properties and operating regimes [41].

AlN and scandium-doped AlN (AlScN) and thin-film PZT are commonly used as the piezoelectric MEMS in microsystems, due to their integration with silicon micromachining. Undoped AlN has a rather small piezoelectric response (typical d33 ≈ 4–6 pm/V) but is highly compatible with CMOS and has low dielectric loss and a high acoustic quality factor. Experimentally, a several-fold (about 3–5x) increase in the values of d33 and e31,f is observed in the experimentally determined values of the piezoelectric response of AlN, with typical Sc concentrations of 20–40%, when Sc doping is used instead of pure AlN, depending on the composition, film orientation and deposition factors. This improvement is specific to the materials and the processes and it must not be taken to be omnipresent. Piezoelectric actuators are of bulk and stack types, with the bulk and stack types most commonly using PZT ceramics with much higher piezoelectric coefficients [42,43].

In bulk PZT, typical values of d33 are in the range of ~300–700 pC/N as a function of a strong dependence on the composition (soft vs. hard PZT) and dopant chemistry as well as poling conditions. These values are typical for bulk ceramics and cannot be directly compared to thin-film coefficients without correction for thickness, geometry and drive voltage [44].

##### Displacement Scaling and Characteristics of Performance

Piezoelectric actuators are defined by a large force density and wide bandwidth, which can sometimes lie within the range of the kilohertz to megahertz band. However, the intrinsic piezoelectric strain is small (usually between 0.1 and 0.2), and this restricts the direct movement of the piezoelectric actuators. In the case of the MEMS scale, displacements have been reported to be between 0.1 and 100 µm, depending on the thickness of the film and the configuration of the electrodes and the voltage delivered. Movements of a few millimeters can only be made by mechanical amplification mechanisms (e.g., lever-type, bridge-type, Moonie, Rainbow, or rhombus actuators), which amplify stroke at the expense of more structural complexity and lower bandwidth [45,46].

##### Applicability to Microfluidic and Acoustofluidic Actuation

Piezoelectric actuators are of special significance in microfluidic systems, because they are prompt in reaction, have a high degree of controllability, and are able to act at a high frequency. Piezoelectric components are commonly applied in direct microfluidic actuation, both in membrane-based pumping and valving, in which applied voltage causes diaphragm deflection to pump or block the flow of fluids [47]. Other than direct mechanical actuation, piezoelectric devices are the basis of acoustofluidic technologies, such as acoustic streaming, acoustophoresis, and surface acoustic wave (SAW) platforms of particle, droplet, and cell manipulation. Incorporated into microfluidics, significant practical limitations are the bonding of the piezoelectric element to the chip, functionality in liquid, dielectric and Joule heating in high drive amplitudes, and the fundamental trade-off between the amplitude of displacement and bandwidth. These factors have a significant impact of the decision that thin-film piezo MEMS or bulk piezo actuators be the part selected in a certain microfluidic or acoustic application [48]. Comparison of the key performance characteristics of various piezoelectric actuation platforms are presented in Table 1.

**Figure 3:**
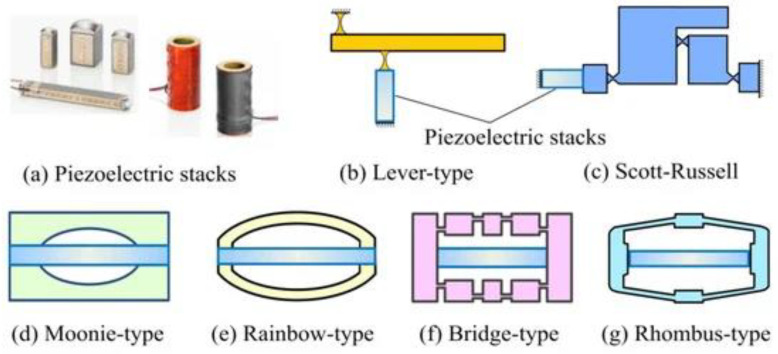
(**a**) Piezoelectric stacks and (**b**–**g**) schematic forms of common types of mechanically amplified piezoelectric actuators by compliant mechanism [52].

Figure 3 represents piezoelectric stack actuators, including lever-type, Scott-Russell, Moonie, Rainbow, bridge-type and rhombus-type piezoelectric actuators.

It is necessary to note that not every piezoelectric actuator is based on the principles of mechanical amplification. Directly actuated piezoelectric devices, such as thin film piezoelectric diaphragms and cantilever-based piezoelectric actuators, obtain their displacement directly by the inverse piezoelectric effect and are widely applied in microvalves, micropumps, and ultrasonic transducers. Mechanically enhanced piezoelectric actuators (e.g., lever-type, bridge-type, Moonie, rhombus, etc.) rely on parts known as compliant mechanisms to use small intrinsic piezoelectric strains (ultra-small displacements) to produce larger output displacements. Although amplification enhances stroke, it brings on extra structural complexities, concentration of stress and bandwidth constraints. Although the direct and mechanically amplified piezoelectric actuation is a matter of choice, it is dependent on the displacement requirements, force output, operating frequency, and the constraint of fabrication [25].

**Figure 4:**
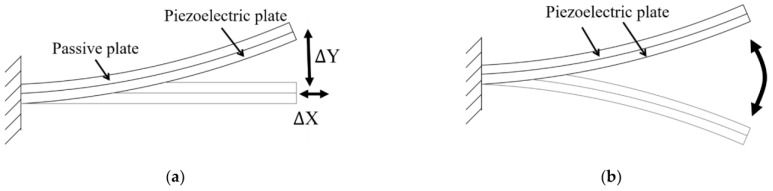
Schematic of (**a**) unimorph configuration and (**b**) bimorph configuration [53].

Figure 4a provides the diagrammatic representation of a unimorph configuration comprising one piezoelectric plate and one passive plate. Two piezoelectric elements may also be made into a bimorph configuration in other applications to generate a two-way bending motion, as shown in Figure 4b.

It must be emphasized that not every piezoelectric actuator is a mechanically amplified actuator. A large number of piezoelectric actuators are designed to act directly in actuation mode, and mechanical amplification is only used in special designs where more displacement is needed.

#### 2.1.4. Electromagnetic Actuators

Electromagnetic actuators produce mechanical movement by the interaction between electric currents and magnetic fields and are appealing to use where higher force output is needed, and also where a linear controllability is desired. The actuation mechanism governing a current carrying conductor or coil in the magnetic field is the Lorentz force, which has to be represented in the form of a vector as

*F = I L × B*

where *I* is the applied current, *L* is the effective conductor length vector within the magnetic field, and *B* is the magnetic flux density. The force magnitude is therefore given by

*F = I L B sin θ*

where *θ* is the angle between the current direction and the magnetic field, expressly referring to the dependence of force on geometry and orientation, not just on current or the strength of the magnetic field [54].

Besides actuation by Lorentz forces, actuation can also be done with electromagnetic microactuators, which can act through magnetic attraction or reluctance forces, whereby movement is caused by the desire of a magnetic or soft-magnetic structure to reduce magnetic reluctance in an applied field. Both of these actuation modes are physically different and result in different design trade-offs regarding force-density, stroke and power consumption: (i) Lorentz-force actuation on current carrying conductors or coils and (ii) magnetic-attraction-based actuation [55,56].

##### Adjusted Performance Scaling and Range

The forces produced by electromagnetic MEMS actuators are typically of the order of a few tens of micronewtons up to a few millinewtons, depending on the geometry of the coils, magnetic field strength and drive current. But in the case of monolithic MEMS structures, the inherent displacement is usually restricted within the range of the micrometer to tens of micrometers. Reports of millimeter scale movements in the actuation of electromagnetism typically relate to macro-scale actuation, mechanically enhanced structures or failure to interpret units. Indeed, electromagnetic microactuators driven at currents of 50–100 mA have been demonstrated to generate displacements on the order of tens to hundreds of micrometers, but not millimeters, when the original device size and measurements are appropriately scaled [57,58,59].

##### Power Heating and Joule Consumption

EM actuators are typically not actuated through thermal expansion, but they typically need fairly large drive currents and thus may cause Joule heating in the conduction coils. This resistive heating may result in a temperature increase, thermal drift and higher power consumption, which encourages high design trade-offs that include coil resistance, duty cycle and heat dissipation. This heating can affect fluid viscosity, can cause a thermal gradient or have a negative impact on some biological samples that are sensitive to temperature, so thermal management is a critical issue when using electromagnetic actuation on-chip [59,60].

##### Application to Microfluidic Internetting

In microfluidic applications, electromagnetic actuation is practical at system scales, based on a number of system-level considerations such as the locations of the magnets, packaging, size of the device, the frequency at which strokes and forces can be achieved with acceptable current levels, the electromagnetic compatibility, and the long-term durability of the system when used in a cyclic mode. Certain reported demonstrations of electromagnetic microactuators, including tunable electromagnetic metamaterials, are not microfluidic actuators, but can be used to illustrate the design and scaling of electromagnetic microactuators. More closely related to microfluidics are electromagnetic membrane and diaphragm actuators, which have been investigated in micropumping, valving and flow control, where greater force output and linear controllability is beneficial despite greater power use than in electrostatic methods [61]. Table 2 summarizes the key performance features of electromagnetic actuation platforms of MEMS based microfluidic systems.

**Figure 5:**
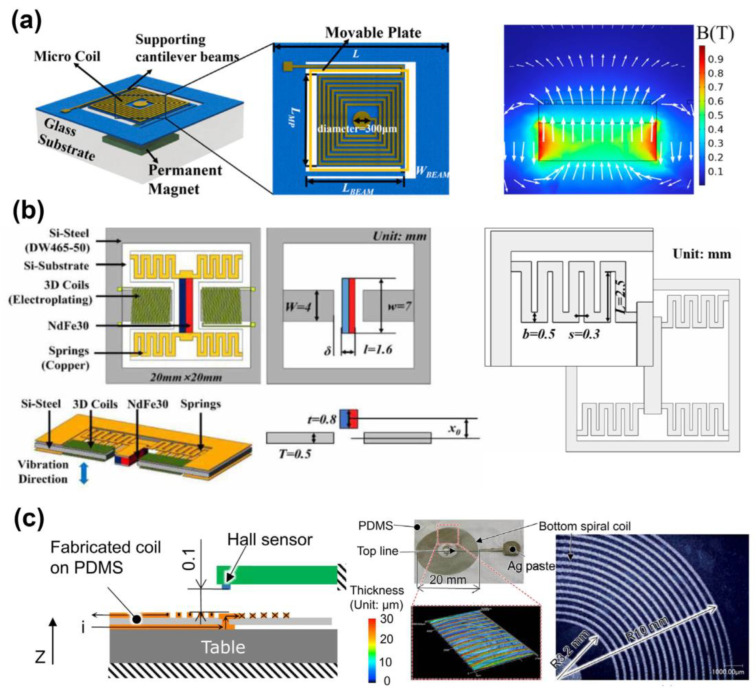
(**a**) Design of the electromagnetic actuator (EMA) and the magnetic flux density generated by a coil, superimposed on a permanent magnet [64]. (**b**) Overall structure of the micro VEH and spring geometric parameters [65]. (**c**) Vertical magnetic flux density generated by the current-carrying coil and photograph of the coil and close-up of a spiral coil [66].

Zhou et al. [64] introduce an adaptable electromagnetic actuator of tunable terahertz metamaterials built of cantilever support beams, an integrated coil on top of the cantilever movable plate, and a permanent magnet on the bottom of the plate to create a fixed magnetic field, as depicted in Figure 5a. The fabrication process is also made easy by the use of flexible polyimide as the substrate material, as opposed to the conventional silicon-based micro machining methods.

Though electromagnetic MEMS actuators are able to produce relatively bigger displacements, with respect to electrostatic devices, displacements had been reported. The typical implementations in microfluidics are tens or hundreds of micrometers. Greater reported motions are usually related to non-microfluidic or meso-scale systems and are thus not representative of microfluidic actuation, based on MEMS.

Figure 5b,c present typical electromagnetic MEMS actuator architectures that show variation in the force-generation mechanisms and fabrication approaches that have been reported in the literature.

Tao et al. [65] create a micro-electromagnetic vibration energy collector that is designed using a 3D MEMS coil and silicon steel sheet core structure, and it is able to optimize the energy collection efficiency and output power, as indicated in Figure 5b. A numerical simulation is applied to analyze the effects of the air gap and starting magnet position offset on the dynamic characteristics and output performance of the system and it is found that there is an optimum initial position offset to obtain the maximum output power. The offered theoretical framework and design approach may be applied to the design and may optimize the devices of a similar structure, suggesting a novel concept and technical path to MEMS electromagnetic vibration energy-harvesting technology. Qi et al. [66] create a flexible coil of soft electromagnetic microactuators (SEMMAs), based on a conductive polymer composite (CPC) of PDMS, which has been considered in portable systems with microfluidics because of the low drive voltage and high response, yet flexibilities have been hampered with membranes consisting of rigid permanent magnets or conductive liquids. They suggest a screen-printing method as a way of printing a coil based on CPC onto a membrane of PDMS that has the capability of maintaining the ability to move the part. The printed CPC coil is of a low Youngs modulus and small thickness, such that it does not contribute much to the stiffness of the membrane. A prototype SEMMA is fabricated and tested with a 19.5 mm thick spiral CPC-based coil on a Φ30 mm × t0.1 mm PDMS substrate, as illustrated in Figure 5c, and it is observed to exhibit both static displacements and asymmetrical displacements to various voltage inputs.

These examples are not proposed to be a comprehensive comparison, but they emphasize the major trade-offs of the force output, complexity of fabrication and integration issues when electromagnetic actuation is used on microfluidic systems.

#### 2.1.5. Surface-Tension/Electrowetting-Based Actuators

Surface-tension or electrowetting-based actuators are based on the electric modulation of the wettability of a liquid droplet on a solid surface. In this case, the technique of applying an external voltage to regulate the apparent contact angle of a conductive droplet placed on an insulating dielectric layer is known as electrowetting on dielectric (EWOD). The basic behavior of EWOD is characterized by the Young–Lippmann equation [67].

The Lippmann–Youngequation

$$\cos\theta_{V}=\cos\theta_{O}-\frac{\varepsilon_{r}\varepsilon_{o}V^{2}}{2\sigma_{lg}h}$$

where $\theta_{V}$ is the contact angle under an applied voltage V, $\theta_{0}$ is the equilibrium contact angle at zero bias, $\varepsilon_{r}$ is the relative permittivity of the dielectric layer, $\varepsilon_{0}$ is the vacuum permittivity, $\gamma_{lv}$ is the liquid–vapor (or liquid–oil) interfacial tension, and h is the thickness of the dielectric layer. This relation is derived under the typical EWOD assumptions of a conductive droplet, an insulating dielectric layer, and quasi-static electrowetting conditions [67].

In the practical implementations, EWOD devices are often designed with low-hysteresis hydrophobic surface coatings (anti-fouling coatings), which are purpose-built to minimize contact-line pinning, droplet sticking and biofouling when accompanied by biological fluids [68,69]. Such surface treatments are extremely important to some of the key characteristics of repeated droplet motion and operational stability in some reconfigurable microfluidic systems [68]. EWOD systems provide programmable droplet movement, splitting and merging regardless of the absence of moving mechanical elements, which provides an accurate control of the droplet volumes in the pL-nL range [70].

The attainable operating window is highly dependent on the device design parameters: the actuation voltage needed is largely controlled by the dielectric thickness and dielectric stack properties; the minimum and maximum droplet volumes are dependent on the electrode pitch and plate separation, as well as the surrounding medium; the characteristic response time, which is often on the order of tens to hundreds of milliseconds, is dependent on the viscosity of the droplets, contact line friction and hysteresis, as well as whether the operation is performed in the air or in oil [70,71].

Despite all these benefits, utilization of EWOD-based actuation has several significant practical limitations. At higher voltages, contact angle saturation limits further modify wettability, which decreases actuation efficiency. Contact angle hysteresis and stick-slip movement due to defects and the chemical inhomogeneity of the surface may degrade the positioning accuracy and repeatability [72].

Long-term operation may also be compromised by charge trapping and dielectric charging and related performance drift, as well as dielectric breakdown under high electric fields [73].

From a design point of view, these limitations can be minimized with proper selection of dielectric materials and thickness, the use of strong hydrophobic or lubricant-infused top coatings, optimal driving waveforms (e.g., AC vs. DC actuation), and operation in oil-filled environments to suppress evaporation and extrusion of hysteresis. Appropriate strategies for surface conditioning and cleaning are also necessary when EWOD devices are used for the repetition of measurements with biological samples [74].

#### 2.1.6. Pneumatic and Hydropneumatic Actuators

Pneumatic microvalves make up one of the most mature, taper UUons, and adopted actuation mechanisms for programmable microfluidic control, in view of their capability to supply on–off trustworthy switching and precise flow metering/a willow route, as well as scalable total routing of complex fluidic networks. In MEMS-scale implementations of these, the most common implementation is pneumatic actuation provided by membrane-based microvalves, in which an externally applied pressure deforms an elastomeric membrane to control the flow in an underlying microchannel [75].

##### Basic Taxonomy and Architecture of Valves

The pneumatic microvalves are generally categorized depending on the membrane configuration and the default valve state. Common architectures of these are normally open and normally closed membrane valves and single-layer and multilayer soft lithography implementations. Multilayer designs, developed first with elastomeric materials such as PDMS, allow for the implementation of the vertical integration of the control and flow layers, and as a result, thousands of valves are incorporated into a single chip footprint. The most important performance parameters for pneumatic microvalves are the response time (usually milliseconds); leakage rate; backpressure tolerance, which could be more than 100 kPa; and fatigue life, when the valve is actuated repeatedly [76].

##### Programmability of Routing and Large-Scale Integration

A characteristic benefit of pneumatic microvalves is the ability to bring them into large-scale integration and programmable fluidic control. By integrating valve arrays with strategies like pneumatic multiplexing, complex routing, metering and sequencing operations can be achieved with a few control lines from outside. This concept of microfluidic large-scale integration (mLSI) allows for the implementation of sophisticated fluidic logic, such as parallel processing of samples, addressable delivery of reagents and the implementation of an automated assay workflow. Such programmability means that pneumatic actuation can be distinguished from the passive, microfluidic architectures in which the flow paths are fixed when they are fabricated [76].

##### Valve-Enabled Droplet Workflows

Beyond continuous-flow control, pneumatic microvalves are also an absolutely essential technology in active droplet microfluidics. Valve-based systems provide the possibility to generate droplets deterministically in terms of user-defined timing and volume and are followed by site-selectively chosen droplet splitting and controlled merging operations, which are not possible with purely passive break-up mechanisms. Several recent papers have now shown combining integrated pneumatic microvalve platforms with intercoupled droplet sizers, programmable splitting ratios and reconfigurable merging sequences to simulate dropwise pipettes, i.e., reduction in the microscopic collection of fluids. Such valve-enabled droplet workflows can be used to support multi-step biochemical protocols, like reagent dosing, serial dilution and reaction chaining within a given microfluidic device [77].

##### Practical Mode of Operation and Limitations

In practical operation, actuation pressures within the range of 20–200 kPa (according to membrane thickness, channel geometry and material properties) are required for pneumatic microvalves [77]. While the use of pneumatic actuation has provided debatable advantages from its robustness and compatibility with low-page iron requirements, the methodology also presents features of a practical trade-off. Pressure sources and control hardware used externally add an extra system footprint and affect portability, whilst the long period of operation can be compromised due to membrane fatigue, leakage or drift in valve response to extensive cycling [75]. Thermopneumatic variants, being based on the thermal expansion of trapped gas, have the disadvantage of being less dependent on external pressure lines, but generally, they are lacking in terms of response time and power consumption. Overall, pneumatic and hydropneumatic actuators are still at the core of programmable microfluidics, especially in the cases where accurate routing, integrated density or deterministic droplet manipulation is needed. Their relevancy is supported by advances in new valve materials, multiplexing strategies and hybridization with other forms of actuation [78].

**Figure 6:**
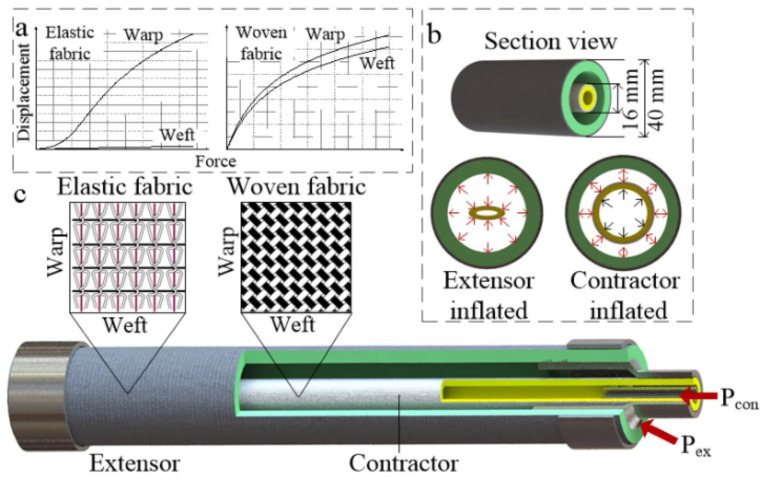
A conceptual representation of the soft pneumatic actuator. (**a**) Force-deformation test of fabrics in warp and weft direction. (**b**) Section view of the actuator and state of extensor and contractor bladders in inflation. (**c**) Overall structure of actuator [79].

The conceptualized representation of the soft actuator is represented in Figure 6. Figure 6a shows the force deformation, i.e., the anisotropic mechanical properties of the elastic and woven fabrics, in the warp and weft directions of the fabric. Figure 6b provides the cross-sectional view of the actuator, where the internal set-up of the actuator and the inflation conditions of the extensor and contractor bladders under pressure are depicted. Figure 6c presents the general structural design of the actuator, including the arrangement of the fabric reinforced layers and position of the extender and contractor chamber vis a vis each other.

##### Benchmarking Perspective

Even though MEMS actuator technologies have been commonly discussed individually, there has been a paucity of direct comparison across the MEMS actuation mechanism because of the variability with which key performance indicators are reported. Table 3 provides an order-of-magnitude benchmarking of the most important parameters applicable to a microfluidic integration that encompass scalability, power consumption, fabrication complexity, and possible force and displacement. The electrostatic actuators are especially appealing to low-power and high-density integration, whereas electrothermal actuators have a higher force, but with a higher efficiency and temperature control cost. Piezoelectric actuators can offer rapid and accurate control and overcome fabrication and material variation issues. Pneumatic actuation is the most scalable of all complex microfluidic networks, but needs heavy off-chip infrastructure. This analogy draws our attention to the fact that the choice of actuators is always application-specific and trade-offs regulate it, rather than the optimum performance [79,80,81].

### 2.2. By Function in Microfluidics

#### 2.2.1. Pumps

Micropumps generate the net fluid flow in a number of ways and recent reviews have included the optimization strategies on performance. There are major types of diaphragm pumps, which operate by reciprocating motion and include check valves; peristaltic pumps, which operate by sequential actuation; and valveless pumps, for which the flow is directed using diffuser/nozzle elements. Both types have their own benefits regarding the accuracy of the flow rates, the ability to work with various fluid types, and backpressure [13].

#### 2.2.2. Valves

Microvalves offer very important fluidic control that has special performance needs, and which come in various modes such as normally open/closed to allow fail-safe operation, latchable valves to allow maintenance of a power-free state, and proportional valves that allow a continuous flow, rather than an on/off mode of operation. The choice is based on the application requirements of response time, leakage rate, and power consumption limitations [82].

#### 2.2.3. Mixers

At low Reynolds numbers, active micromixers are used to overcome diffusion-based limiting of mixing by various actuation mechanisms such as acoustic streaming of SAW-based vigorous mixing, magnetic stirring with rotating magnetic elements and electrokinetic instability in which chaotic advection is induced. These techniques are also quite efficient when mixing in comparison to passive methods, which allows for a rapid homogenization of reagents to be used in chemical and biological analyses [83].

#### 2.2.4. Selective and Programmable Assay “Pipetting-on-Chip” Modified by Microvalves

A distinctive and vital practical differentiation of compositions in microvalve-based droplet microfluidic systems is their opportunity to perform selective, event-based operation on individual droplets. Unlike the passive droplet architectures where droplet splitting, merging and routing is only function of channel geometry and flow conditions, the integrated microvalves facilitate deterministic and programmable control, and operations can be performed on select droplets while leaving other droplets unaffected. This selective capability is especially relevant in the case of biological and chemical high-throughput screening, where conditional choice and manipulation of samples are needed for the efficient execution of the assay [77,84]. Amazingly, by actuating valves based on timing and/or droplet position or externally measured signals, microvalve-based systems can allow selectivity for splitting, merging, injecting reagent, and routing of droplets. Collectively, these functions implement a form of “pipetting-on-chip,” whereby droplets are the isolated reaction vessels corresponding to wells in a microtiter plate, but with the added benefit of automation, reconfigurability, and operation at picoliter to nanoliter volumes. Such programmable control allows for combinatorial reaction permutations, on-demand sampling, and automatic multi-step assay workflows such as branching assay trees that can be difficult or impossible to accomplish by only using passive droplet systems [85,86].

##### Selective Droplet Breaking Triggered by Valve Actuation

Selective droplet splitting is a good example of a clear and representative representation of valve-enabled programmability. In valve-actuated bypass or junction geometries, the local hydraulic resistance can be dynamically changed, determining split or diversion, volume disconnect, and so on. Raveshi et al. [85] demonstrated selective droplet splitting by single layer microfluidic valves, in which the actuation of a pneumatically deformable valve at the inlet of a bypass channel allowed for controlled splitting of a selected “mother” droplet, whilst non-selected droplets passed through the main channel without breakup. Importantly, the split ratio was adjustable by setting the valve actuation pressure, thus making it possible to generate daughter droplets with prescribed volumes. Building on this concept, Agnihotri et al. [86] achieved selective on-chip droplet splitting at a variety of locations by integrated microvalves, which allows the redistribution of droplet contents along different downstream routes. Together, these studies show how the microvalves enable the switching of droplet splitting from a passive process defined by the geometrical shape to an addressable and programmable process as a basis for on-chip liquid handling functions of similar character to selective pipetting.

##### Relevance to Screening Style and High-Throughput Assay Workflows

The ability to selectively manipulate individual droplets on the scale of a single droplet directly supports screening-style workflows, similarly to those undertaken with the use of microtiter plates, but at the droplet scale. In high-throughput biological screening, droplets containing samples of interest can be selectively processed for example, separated for parallel analysis, combined with additional reagents, or directed to specific detection modules and other irrelevant droplets will be bypassed or discarded. Brouzes et al. [84] showed the benefit of droplet microfluidic technology in high-throughput single-cell screening technology via compartmentalizing single cells and reactions in droplets and processing them in parallel. The integration of microvalves is a further step towards this paradigm, since it allows for the conditional and programmable assay progression (not uniform processing of all droplets). Overall, microvalve-enabled selectivity takes droplet microfluidics from a passive transport platform to a programmable microfluidic processing device that is able to execute complicated decisional execution workflows. This selective “pipetting-on-chip” capability is a major practical strength of the use of valve-based droplet platforms for applications related to single-cell analysis, combinatorial chemistry and automated high-throughput screening [84,85,86].

#### 2.2.5. Droplet Generators and Manipulators

In addition to EWOD, active droplet generation offers a fine control over size using flow-focusing with active control, T-junction with piezoelectric actuation and acoustic droplet ejection. The techniques allow us to create highly monodisperse droplets of a controllable size and frequency, which are needed in digital microfluidics or compartmentalized reactions [87].

#### 2.2.6. Actuators/Drug-Delivery Microneedles

In more complex versions of microneedles, fluidic components are incorporated, such as smart patch systems, closed-loop drug delivery platforms, and even biodegradable implants. These systems signify the overlap of the microfluidic actuation and transdermal drug delivery and the therapeutics are delivered in a very controlled and precise manner with minimal intervention on the part of the patient [18,88].

#### 2.2.7. Micromechanical Sensors in Coupling with Actuators

Closed-loop control allows for fine fluidic control using implementation plans such as in-built pressure/flow sensors, real-time feedback algorithms, and machine learning controlled systems. This is the most advanced form of sophistication in a microfluidic system which can execute autonomous functions and is able to counterbalance environmental fluctuations and system wear [89].

### 2.3. According to Operational Scale and Force Requirements

#### 2.3.1. Nano-Scale Actuators

Nano-scale actuators are actuators that work on a sub-micrometer and molecular length scale and take advantage of the electroosmotic flow, dielectrophoresis, and optical trapping phenomena to figure out and precisely handle nanoparticles and biomolecules. Usually, these mechanisms produce forces in the pico-Newton to nano-Newton scale, and the scale of displacement can only usually reach the nanometer to sub-micrometer range (<1 μm). These properties of force displacement find use in applications such as the manipulation of DNA, assembly of nanoparticles and highly specific sorting of molecules [90].

#### 2.3.2. Micro-Scale Actuators

MEMS actuator devices are micro-scale in nature (usually on a scale of several micrometers to sub-millimeters) and are designed and manufactured using standard processes of microfabrication, usually alongside microfluidic systems. Microfluidic pumping, valving, mixing, and manipulation of the particles or cells are most often actuated by electrostatic, piezoelectric and electrothermal methods. Micro-scale actuators using MEMS normally produce micro-Newton to milli-Newton range forces, depending on the actuator’s geometry, material and environment. The displacement that can be realized is usually restricted to within the nanometer to tens of micrometers, unless some external mechanical amplification processes are used. Such properties render MEMS actuators to be appropriate for the fine control of microfluids, as opposed to large-scale movement [23,91,92].

### 2.4. By System Integration Level

#### 2.4.1. Discrete Component Actuators

Discrete actuators have become isolated elements of microfluidic systems, which provide design flexibility and simple replacement. They are individual piezoelectric disks, individual electromagnetic coils and discrete thermal components, which may be deposited into custom fluidic systems. As much as they have flexibility in their designs, they tend to be problematic in interconnecting and miniaturizing systems [93].

#### 2.4.2. Semi-Integrated Actuators

Partially integrated systems are systems that integrate actuators and simple control electronics, but still some external components. Examples are micro-pumps that have embedded drivers and external control units and valve arrays that have on-chip sensing and off-chip processing. It is a method that balances performance and manufacturing complexity, which is the reason why it involves low volume manufacturing and prototyping [94].

#### 2.4.3. Fully Integrated Systems

Microfluidic actuators are fully integrated to include sensing, actuation, control, and in some cases, power management on a single chip. These systems are the state of the art in MEMS technology and allow for fully self-contained lab-on-chip systems. They have been used in autonomous chemical sensors, implantable medical devices, and portable diagnostic platforms that need minimum external elements [95].

### 2.5. Via Application-Specific Requirements

#### 2.5.1. Biomedical and Clinical Actuators

The design of biomedical actuators is characterized by high demands in the terms of biocompatibility, sterilization performance, and reliability. The materials should be of ISO 10993 standards, and the designs should be such that biological fluids do not clog or degrade them. These actuators drive the essential equipment like insulin pumps, chemotherapy drug delivery systems and in vitro diagnostic equipment, where failure may prove to be devastating [96].

#### 2.5.2. Research and Development Actuators

Research and development (R&D) actuators are mostly used in experimental and laboratory applications to investigate new actuation modes, new materials, and system-level integration strategies, but not to be used on a long-term commercial basis. These actuators are important in demonstrations of proof-of-concept and early-stage performance of microfluidic systems.

Piezoelectric actuators are considered to be one of the most commonly studied in R&D settings because of their quick response and the ability to control the displacement precisely. The example diaphragm-type and cantilever-based piezoelectric actuators are already in place in micropumps and microvalves on the laboratory scale. The most common displacements reported are 1–10 um, and the response time is within the sub-millisecond through milliseconds range; thus, it can be used in droplet generation, biochemical assays and controlled cell manipulation [97].

Electrothermal microactuators are also actively investigated in research works where more force output is needed. These actuators usually offer displacements of the order of micrometers, together with forces of the order of tens to hundreds of micro-Newtons at the expense of higher power usage and slower thermal response. These properties render the electrothermal actuators suitable in experimental research into valve sealing, particle trapping, and the microgripper-based manipulation [98].

Moreover, magnetic and soft polymer-based actuators are currently being actively developed in the R&D environment, as they can be used in an application where it is necessary to have high deformation and mechanical compliance. The displacements attained using these actuators can be in the tens to hundreds of micrometers, with quite low actuation fields opening up possibilities for bio-inspired actuation, soft robotics, and adaptive microfluidic designs [99,100].

Altogether, R&D actuators can be considered as the necessary platforms to conduct quick prototyping, systematic performance testing, and experimental validation of novel actuator concepts before their conversion into microfluidic technologies that can be scaled to be robust and scalable [101].

## 3. Materials and Fabrication Techniques

The choice of suitable materials and fabrication techniques play an important role in the development of high-performance MEMS actuators to work with microfluids. The material systems and manufacturing technologies that will permit the functional microfluidic devices to become possible are comprehensively considered [102]. It will provide a general overview of the advanced materials and fabrication methods employed in MEMS microfluidic systems, the major structural and functional materials employed, the major microfabrication processes, and their combination into multilayer microfluidic devices comprising embedded actuation, sensing and fluidic interfaces.

### 3.1. Structural Materials

#### 3.1.1. Silicon-Based Materials

Single-crystal silicon is the most popular substrate material, because it possesses good mechanical characteristics and long-established microfabrication processes. Silicon-on-insulator (SOI) substrates offer improved operations of devices by improving electrical isolation and the manufacture of more complex structures. Porous silicon substrates have allowed for special fluidic characteristics by controlling the surface geometry and increasing the surface area to be useful in biology [103].

**Figure 7:**
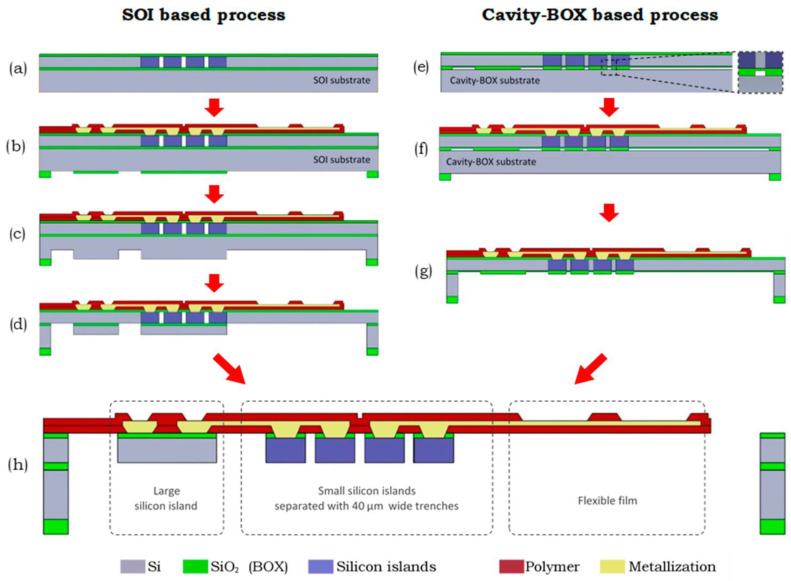
Flow charts of the simplified manufacturing process of the semiflexible DBS device through the trench-based F2R technology versus through the cavity-BOX substrate. Left (**a**–**d**): SOI-based process, sealed trenches on the front side of the wafer, and limited to a two-step backside etch process. Right (**e**–**g**): Cavity-BOX process with hard-etch mask patterned BOX (patterned to act as etch-stop) as an etch-stop layer. Bottom (**h**): Finished device [104].

A comparison of simplified manufacturing process flows of semiflexible deep brain stimulation (DBS) probes produced by a conventional SOI-based trench process and cavity-BOX-based process is presented in Figure 7. The cavity-BOX approach allows for the creation of foldable DBS structures by monolithic fabrication for DBS, as it exploits the patterned buried oxide for use as an etch-stop and hard etch mask that reduces the process’ complexity and enhances the structural flexibility of the fabricated structures in the final DBS.

#### 3.1.2. Glass and Ceramic Substrates

**Figure 8:**
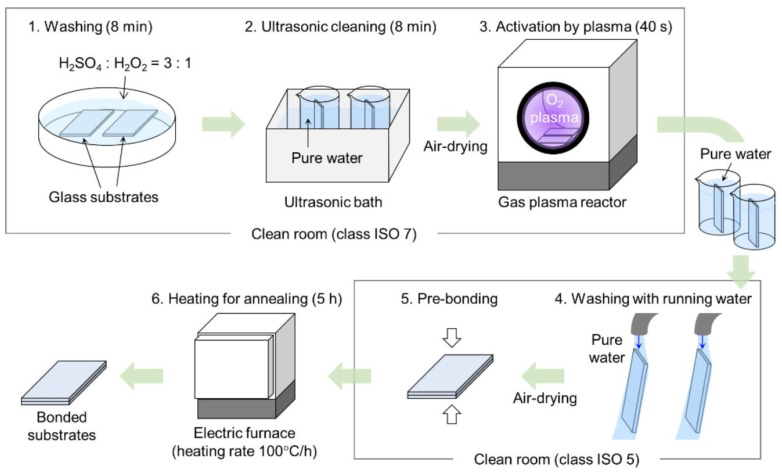
Schematic diagram of low-temperature bonding process for glass substrates utilizing typical facilities in clean rooms [106].

The borosilicate glasses find application due to their optical transparency, chemical resistance and excellent electrical insulation characteristics. Fused silica is better in optical properties and thermal allotment in a high temperature. High-tech ceramics such as alumina and zirconia are exceptionally strong in a mechanical sense and have the chemical inertness to operate in harsh environments [105]. The key process flow of a low-temperature glass substrate bonding process to make MEMS and microfluidic devices is presented in Figure 8.

#### 3.1.3. Conventional Polymer Materials

**Figure 9:**
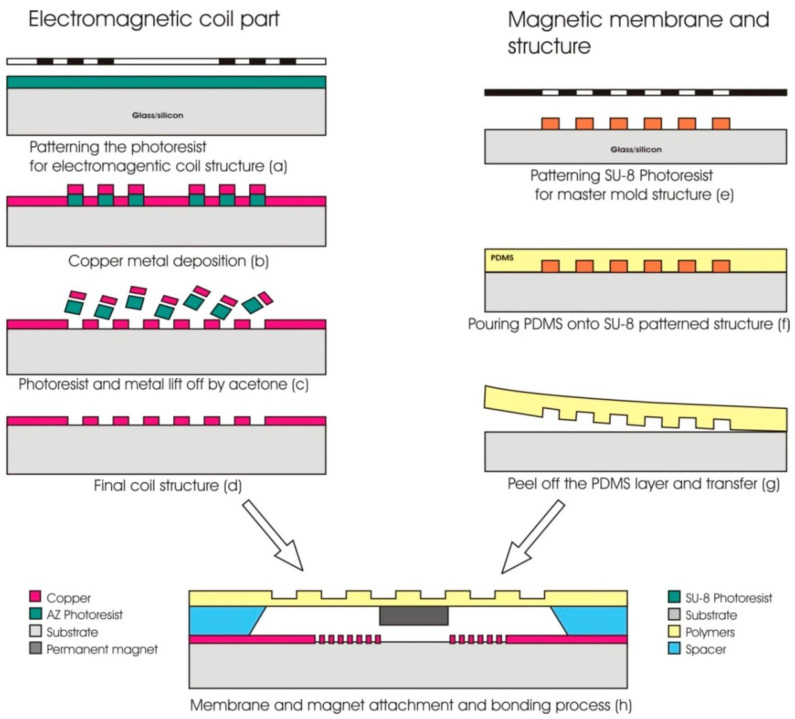
Schematic process steps for the fabrication of polymer-based MEMS electromagnetic actuators with micro-pillar structures using the soft lithography technique: (**a**) patterning of the photoresist for the electromagnetic coil structure; (**b**) copper metal deposition; (**c**) photoresist and metal lift-off by acetone; (**d**) final coil structure; (**e**) patterning of SU-8 photoresist for the master mold; (**f**) pouring PDMS onto the SU-8 patterned structure; (**g**) peeling off the PDMS layer and transfer; and (**h**) membrane and magnet attachment and bonding process [62].

The soft lithography has transformed rapid prototyping by using polydimethylsiloxane (PDMS), which is very flexible and permeable to gases. SU-8 is an epoxy-based negative photoresist that can be used to produce high aspect ratio structures of mechanical components. PMMA and polycarbonate (PC) are superior in their ability to remain mechanically stable, and allow for detection and imaging as they are optically clear [107].The primary fabrication stages of a polymer-based electromagnetic MEMS actuator with micro-pillar designs under soft lithography are presented in Figure 9.

#### 3.1.4. Engineering Thermoplastics

Polyether ether ketone (PEEK) is highly resistant to chemicals and thermal stability in microfluidic applications, which demand thermo-resistance. Cyclic olefin copolymer (COC) offers low water absorption and high optical characteristics in the case of analytical devices. These materials are especially excellent in the case of disposable microfluidic devices because they can be produced in large quantities and have reliable material characteristics [108].

#### 3.1.5. Emerging Flexible Substrates

Paper substrate has become a low cost, ultra-low-cost device platforms of point-of-care diagnostic devices. PET and polyimide allow for the creation of microfluidic systems to be worn. Single-use devices could be produced using eco-friendly biopolymers such as polylactic acid (PLA) and cellulose derivatives [109].

#### 3.1.6. Smart Structural Materials

Shape memory polymers allow for structural modification of the polymers in a programmed manner in response to thermal stimuli. Self-healing materials undergo automatic repair of mechanical damage to increase the life of the device. Adaptive mechanical characteristics with tunable microfluidic operation are achieved with a stimuli-responsive composite [110]. Table 4 outlines the main mechanical, thermal and biocompatibility characteristics of the most popular structural materials used in MEMS microfluidic actuators, their common uses and restrictions.

### 3.2. Functional Materials

#### 3.2.1. Conventional Piezoelectric Materials

**Figure 10:**
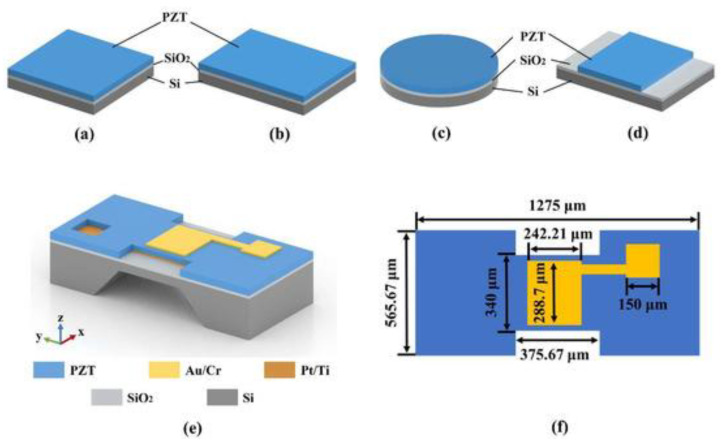
Schematic structure of pMUT’s composite diaphragm with (**a**) square; (**b**) rectangular; (**c**) circular; (**d**) I-shape; (**e**) schematic structure of the pMUT with I-shaped composite diaphragm; and (**f**) structural parameters of the I-shaped diaphragm [115].

The piezoelectric material lead zirconate titanate (PZT) is the most common and is used because of its high piezoelectric coefficient. When creating a lead-free alternative for biocompatible applications, barium titanate (BTO) provides decent performance with no cost for lead. These materials offer the much-needed electromechanical connection to precise fluidic control and sensing [114]. Figure 10 demonstrates improved pMUT frequency and sensitivity. A pMUT with square (Figure 10a), rectangular (Figure 10b), and circular (Figure 10c) composite diaphragms was designed to achieve high resonant frequency and sensitivity while maintaining the same diaphragm area. An I-shaped PZT-based composite diaphragm was subsequently proposed (Figure 10d), and the corresponding pMUT structure and design parameters are shown in Figure 10e,f. The I-shaped PZT film was formed by selectively etching a portion of the diaphragm’s piezoelectric layer to enhance resonant frequency and sensitivity.

#### 3.2.2. Thin-Film Piezoelectric Materials

CMOS-compatible piezoelectric Aluminum nitride (AlN) has high temperature stability. Zinc oxide (ZnO) has excellent piezoelectric performance and comparatively simple deposition mechanisms. The piezoelectric coefficients are greatly improved by using scandium-doped aluminum nitrate (AlScN) and are still capable of being fabricated [116].

Using a Veeco Dektak 150 profilometer (Veeco Instruments Inc., Plainview, NY, USA) and a Keithley model 2400 sourceMeter (Keithley Instruments Inc., Cleveland, OH, USA), the four-point technique was used to determine the films’ electrical resistivity and thickness, respectively. A Bruker model D8 ADVANCE diffractometer (Bruker AXS GmbH, Karlsruhe, Germany) with Cu Kα radiation (λ = 1.5418 Å) (40 kV–40 mA) was used to measure the structural characteristics.

**Figure 11:**
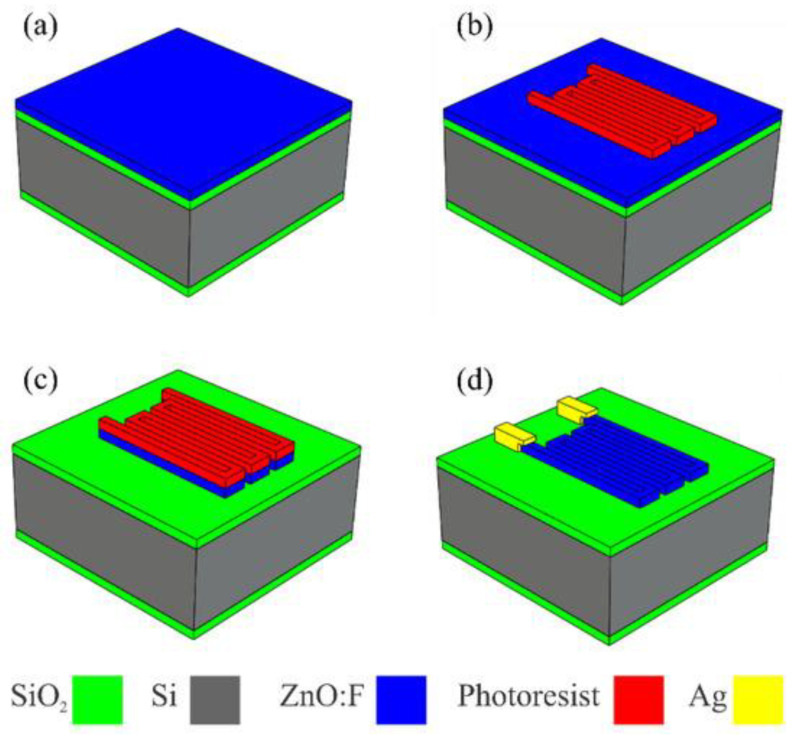
Piezoresistor manufacturing process: (**a**) deposition of a ZnO:F thin film via ultrasonic spray pyrolysis; (**b**) photoresist deposition with spin coating and piezoresistor pattern formation with lithography; (**c**) chemical etching with H_3_PO_4_ + CH_3_COOH + H_2_O (1:1:50) for resistance formation; and (**d**) placement of conductive Ag paste contacts (Sigma Aldrich 735825-25G, resistivity 10−5 Ω.cm) [117].

The device’s construction begins with a rectangular Si (100) substrate covered in an electrically insulating layer of SiO_2_ that is 1 µm thick. Lithography and chemical etching methods were employed to create the ZnO:F piezoresistor, and then contacts were made using conductive silver paste (Sigma-Aldrich, St. Louis, MO, USA; product no. 735825, resistivity 10−5 Ω.cm), as illustrated in Figure 11. It also displays the ZnO:F piezoresistor’s design, as well as the substrate’s measurements [117].

#### 3.2.3. Electroactive Polymer Materials

Ionic polymer–metal composites (IPMCs) allow big deformations of bending with low operating voltages. Dielectric elastomers offer a huge area expansion with the action of electrostatic forces. Polymers such as polyaniline and polypyrrole conductors allow electrically controlled volume changes that can be used in microactuation [118].

**Figure 12:**
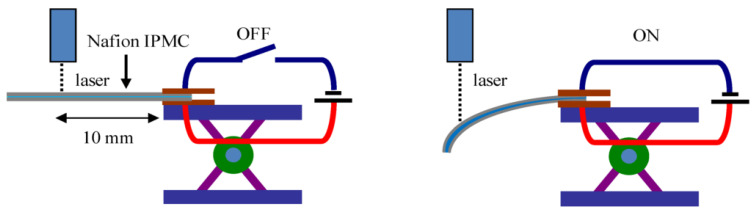
Experimental setup for measuring the tip displacement of Nafion Ionic Polymer–Metal Composite (IPMC). Illustrations on the left and right, respectively, show the shape of Nafion IPMC in the OFF and ON stages [119].

Figure 12 illustrates how a dehydrated Nafion IPMC was clamped at one end to two electrodes that were connected to a power source. The Nafion IPMC bends toward the anode when the voltage is applied. A laser displacement sensor (LK-085, precision 3 μm, KEYENCE, Osaka, Japan) is used to track the position of a point on the strip 10 mm from the clamp point, applied as a rectangular wave (amplitude = 2 V, frequency = 0.05 Hz). Simultaneously, a K-type thermocouple and a Z2000 (HIOKI, Nagano, Japan) humidity sensor were used to record the ambient temperature and relative humidity. Absolute humidity was determined from these. A mathematical conversion of the Nafion IPMC’s measured tip displacement to bending curvature C was performed [119].

#### 3.2.4. Magnetic Functional Materials

Soft magnetic properties can be offered by electroplated Permalloy (NiFe). Neodymium–iron–boron (NdFeB) alloys have good permanent magnetization of micro-scaled geometries. Ferrofluids allow for special control of fluids to be achieved by magnetically manipulating the magnetic field [55,120].

#### 3.2.5. Conductive and Electrode Materials

Microelectrodes can be made of gold and platinum, which offer high stability and conductivity to the electrical environment. The transparent conductive oxides such as indium tin oxide (ITO) allow the electrical activity to be transparent and allow for optical accessibility. Conductive polymers like PEDOT:PSS are mechanically flexible and can be patterned [81,121].

#### 3.2.6. Smart Responsive Materials

Stimuli-responsive hydrogels have a reversible change in volume with respect to environmental stimuli. Molecular alignment liquid crystal elastomers can be programmed to deform. Thermoresponsive polymers such as poly(N-isopropylacrylamide) allow temperature-activated microfluidic functions [27,122]. Table 5 provides a summary of some of the most widely used MEMS actuator materials, their main properties, actuation principles, strengths, weaknesses and applications.

### 3.3. Fabrication Workflows

#### 3.3.1. Bulk Silicon Micromachining

Anisotropic silicon etching with specific dependence on crystal orientation is possible using wet chemical etching with potassium hydroxide (KOH). Another silicon etchant that is CMOS compatible is Tetramethylammonium hydroxide (TMAH). Electrochemical etching methods make porous silicon structures with desired morphology in special applications [126].

#### 3.3.2. Surface Micromachining Processes

Complex multi-layer mechanical structures: The micromarketing of polysilicon surfaces by surface micromachining provides the ability to make multi-layer mechanical structures by the use of sacrificial silicon dioxide layers. Surface micromachining on metal bases makes use of electroplated microstructures, which use photoresist sacrificial layers. Under these methods, suspended mechanical components and integrated microsystems can be made [127].

#### 3.3.3. Advanced Etching Techniques

Deep reactive ion etching (DRIE) allows for a high aspect ratio of silicon with vertical sidewalls. Xenon difluoride (XeF_2_) vapor phase etching offers a high selectivity in silicon etching without etching other materials. Cryogenic etching technology enhances sidewall and control of processes over critical features [128].

#### 3.3.4. Soft Lithography Methods

Microfluidic channels can be prototyped in the sub-micrometer resolution, using replica molding with PDMS. Microcontact printing allows for selective patterned surface modification of biological functionalization. Capillary micromolding has been offered as an alternative method to polymer microstructure creation [107].

#### 3.3.5. Polymer Microfabrication

Hot embossing is a process used to form the microstructure of thermoplastics by thermal pressing onto master molds. High-volume manufacturing of complex geometries of plastic microfluidic devices can be done through injection molding. UV embossing is a variant of thermal embossing and photochemical embossing with an improved resolution and aspect ratio [129].

#### 3.3.6. Additive Manufacturing Approaches

Stereolithography (SLA) offers high-resolution 3D printing of microfluidic devices by photopolymerization. Two-photon polymerization facilitates the feature sizes of complex 3D microstructures of sub-diffraction dimensions. Multi-material fabrication and integration of functional materials is possible with direct ink writing [130]. Table 6 provides a comparison of the regularly used fabrication methods of MEMS microfluidic actuators, based on their attainable feature size, aspect ratio, material compatibility, throughput and relative cost.

### 3.4. Integration and Packaging

#### 3.4.1. Permanent Bonding Techniques

Thermal fusion bonding is a technology that offers high-temperature silicon–glass interfaces. In anodic bonding, permanent bonding is formed between the silicon and glass substrate by the means of an electrostatic field. Epoxy and acrylic resin adhesive bonding is a versatile type of bonding of a variety of materials [112].

#### 3.4.2. Reversible Sealing Methods

Surfaces that have been treated, such as through plasma activation, allow for reversible bonding of PDMS. Experimental and prototype reusable sealing is offered with mechanical clamping systems. Temporary bonding of devices in testing and configuration is provided by pressure-sensitive adhesives [111].

#### 3.4.3. Surface Modification Techniques

The surface wettability and bonding compatibility are improved by using plasma treatment by chemical activation. Self-assembled monolayers (SAMs) offer molecular surface functionalization at a molecular level to particular applications. This can be done using bio-conjugation to immobilize biomolecules [133].

#### 3.4.4. Biocompatibility Enhancement

PEG coatings are used to decrease protein adsorption and cell adhesion so that the surface becomes more biocompatible. The bio-inspired surface modification imitates natural surfaces in order to regulate biological interactions. The ultra-low fouling surfaces are attained by using zwitterionic polymer coatings to allow for long-term biological uses [134].

#### 3.4.5. Fluidic Interfacing Solutions

Microfluidic ports and connections allow for fluidic connections with other systems that are reliable. The embedded interconnects give a smooth passage between the various size scales. Connection systems are self-aligning, which means that they enable people to use the gadget without any complications and to replace it [135].

#### 3.4.6. Reliability Engineering

The predictive life of a device is obtained by using accelerated life testing through the application of controlled stress. Design improvement is achieved by failure mode analysis to identify the reliability problems that are critical. Environmental testing is used to test performance in realistic operating conditions, such as temperature, humidity and mechanical stress [20,136]. A summary of the typical integration and packaging approaches to MEMS microfluidic actuators is provided in Table 7, which compares the bonding forces, thermal and chemical durability, reuse capability, and common areas of use.

## 4. Emerging Design Strategies for MEMS Actuators

New design approaches enable the rapid development of MEMS actuators to support microfluidic applications because of the complicated requirements of the present day microsystems. This area examines the novel design, approaches that are advancing the boundaries of performance, and the integration and functionality of the microfluidic MEMS actuators [11].

### 4.1. Miniaturization and Integration in Lab-on-Chip and Point-of-Care Systems

#### 4.1.1. System-Level Integration Strategies

The shift to have fully integrated LoC systems demands high levels of integration and thus, on a single substrate, there are a variety of actuators. Lately, there have been designs for electrostatic micropumps and piezoelectric valves, as well as thermal mixers in space-constrained devices [138]. With monolithic integration techniques, actuators with fluidic channels can be co-fabricated. This decreases the amount of dead volumes and enhances reliability. Heterogeneous identity is used to integrate silicon-based actuators and polymer microfluidics, leveraging the capabilities of the various materials [93].

#### 4.1.2. Optimization of Point-of-Care Devices

The aim of designing in point-of-care (POC) applications is for reliability, low-power consumption and simple production. New POC-based actuators have fail-safe operations, such as normally closed piezoelectric valves to prevent the leakage of fluids when the power goes off. Energy-efficient designs have sleep mode and energy harvesting capabilities, which increases the life span of the portable design. Focus on disposability employs low-cost, low-complicated methods of fabrication without compromising performance [125].

#### 4.1.3. Multi-Functional Actuator Architectures

New designs contain actuators that incorporate multiple activities, including the piezoelectric elements, which are capable of pumping fluid and detect pressure variations simultaneously [79]. The reconfigurable arrays of actuators can change fluidic protocols dynamically and provide optimal performance with a range of operating conditions. The close-loop control can be enhanced with real-time feedback provided by integrated sensing and actuation, such that it is more accurate and reliable in carrying out a complex fluidic task [139].

**Figure 13:**
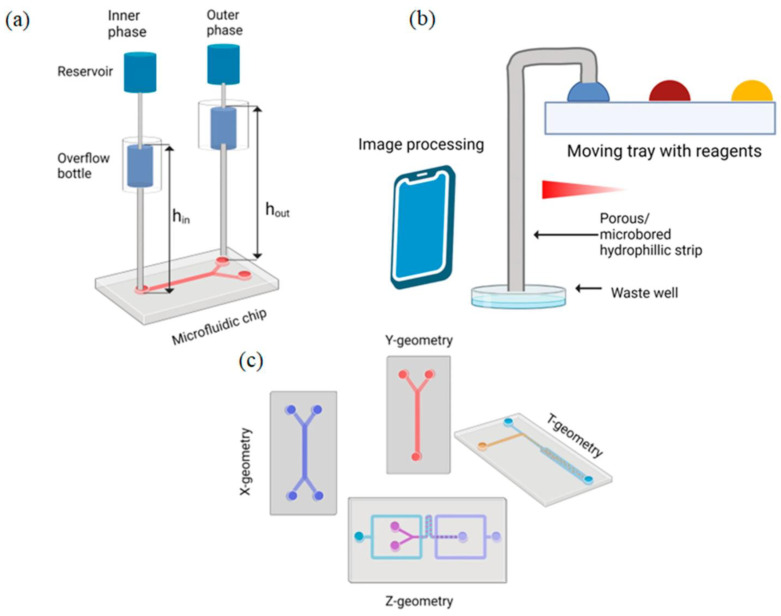
Schematic illustrations of gravity-driven systems: (**a**) semi-open gravity-driven overflow microfluidic flow supply system; (**b**) gravity-driven microfluidic siphon; (**c**) stand-alone pressure-driven 3D microfluidic chemical-sensing analytic device with different channel geometries [139].

Figure 13 provides a a representative system-level diagram to be used in the comparison of passive and gravity-based control of microfluidic flow with actively actuated MEMS-based techniques. The schematics of the microfluidic flow supply system are presented in Figure 13a. By using a two-phase flow interface, the flow stability was checked against a syringe pump system through monitoring the flow interface of two phases that had a low interfacial tension. One of the working principles of the device is depicted by Figure 13b. The POC devices can be developed based on the concept of siphons redirected into a new generation of power-free flow driving techniques. The geometry of the microfluidic channels is depicted in Figure 13c. The authors compared the rates of the fluid flowing under various geometries of the channels and demonstrated that an independent pressure-driven microfluidic apparatus has a high chance of being applied as POC diagnostics [139].

Although this can simplify the system design and decrease the system power requirements, these methods do not provide the programmability, time resolution and closed loop control offered by MEMS actuators and the importance of active actuation in more complex microfluidic systems is evident.

Along with the gravitational flow, there are a number of alternative power-free and passive-based flow generation techniques that are extensively applied in microfluidics. The capillary-based flow sensitizes the surface tension and wettability to take advantage of the autonomous transport of fluids through microchannels, even in the absence of external actuation, and is especially appealing to disposable and point-of-care diagnostics. Another passive strategy is vacuum- or negative-pressure-driven flow, in which a hollow that is pre-evacuated or has contained a known quantity of gas, or load to which a vacuum has been applied, causes the flow of a fluid by driving both sides of the chamber towards an equilibrium pressure that is lower than the original operating fluid pressure; thus, controlling the transfer of fluid into or out of the chamber without any pump is required. These passive flow techniques combined with gravity-based systems provide easy and efficient solutions to low-power microfluidics.

### 4.2. Optimization of Topology and Bio-Inspired Geometries

#### 4.2.1. Computational Topology Optimization

The optimization algorithms of topology have transformed the MEMS actuator design, providing the generation of geometries that optimize performance metrics but keep within the constraints of fabrication. Recent applications take into account multi-physics goals, electromechanical coupling efficiency optimization, fluid–structure interaction and thermal management, all together [140]. Level-set and density-based algorithms create highly non-intuitive geometries, which are better than designs in displacement, force production, and energy consumption [141].

**Figure 14:**
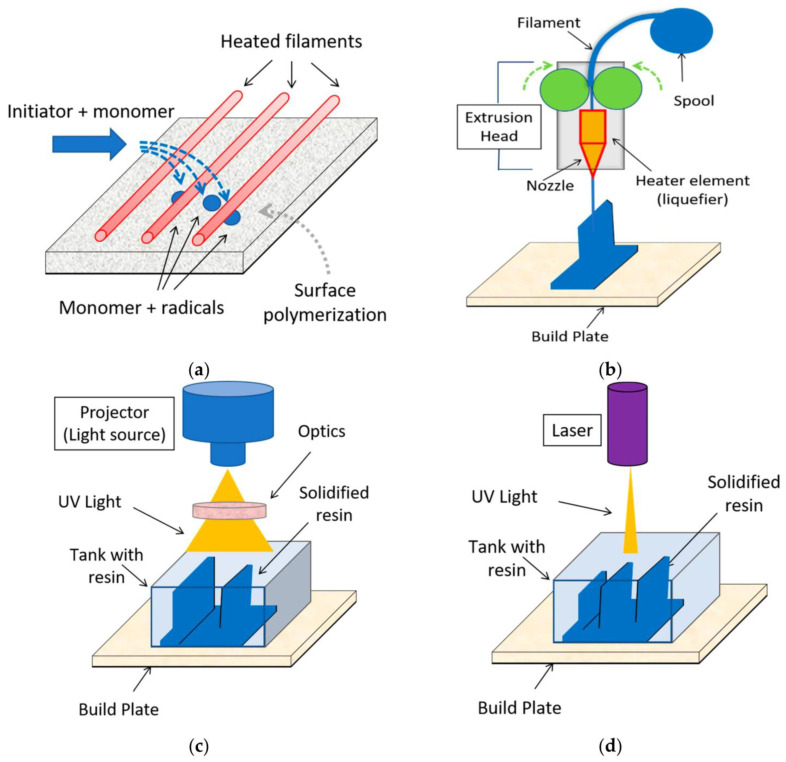
Sketch of the (**a**) initiated CVD, (**b**) fused deposition modeling, (**c**) digital light processing, and (**d**) stereolithography techniques used to prepare the SMPs [142].

In Figure 14a–d, the initiator material(s), along with the monomer(s), is put into the vacuum chamber, whereby these interact with the heated filaments [142].

#### 4.2.2. Principles of Bio-Inspired Design

**Figure 15:**
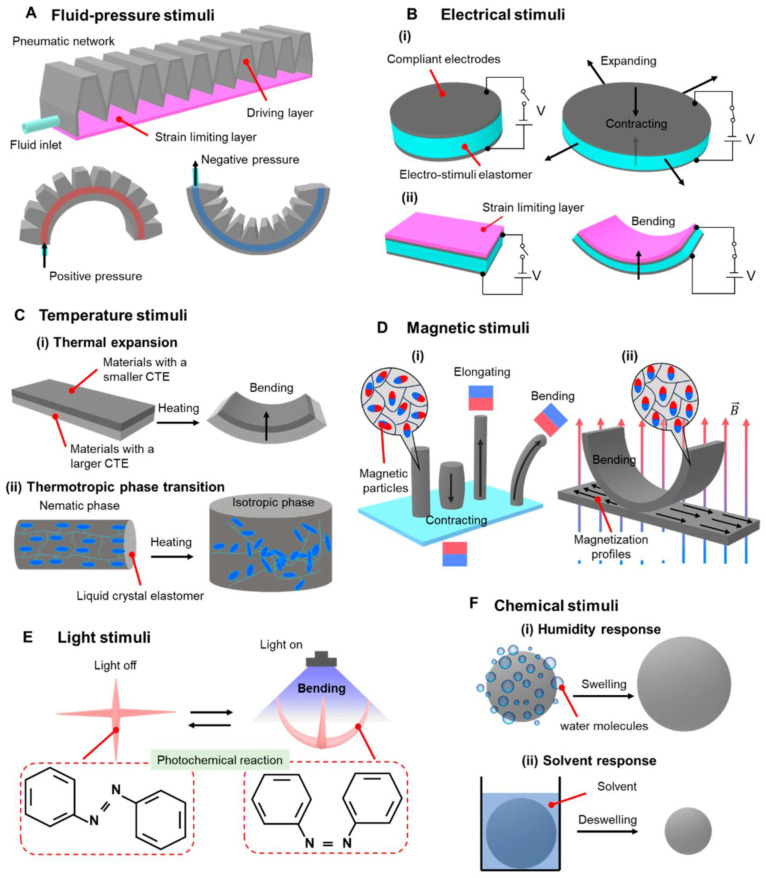
Stimuli-responsive principles of biomimetic soft actuators. (**A**) Pneumatic network actuators are actuated by positive-pressure or negative-pressure gas. (**B**–**F**) Actuation mechanisms triggered by external stimuli: electric fields (**B**), temperature (**C**), magnetic fields (**D**), light (**E**) and chemical stimuli (**F**) [145].

Nature-based designs make use of the principles of evolutionary optimization to produce very efficient geometries of actuators. Compliant mechanisms based on a silk spider web can offer large displacements with a low stress concentration [143]. Principles of nastic plant movement are incorporated into designs of humidity-responsive actuators that do not require externally available power. The valveless pump models have been inspired by a biological pump mechanism like the human heart, in that they can be more efficient as well as less prone to clogging [144]. Figure 15 demonstrates the stimulus response mechanisms of biomimetic soft actuators, which demonstrates actuation by the pneumatic pressure and electrical, thermal, magnetic, optical and chemical stimuli, resulting in controlled bending and deformation.

#### 4.2.3. Fractal and Hierarchical Structures

Fractal geometries also permit actuators in which the surface area–volume ratios are maximized, with better thermal and electrochemical characteristics [146]. Compliant hierarchical mechanisms allocate the stresses in a more even manner, boosting fatigue life and maximum displacement. Multiple-scale self-similar designs offer the benefit of having multiple actuation paths, which enhance reliability due to graceful degradation and not catastrophic failure [147].

### 4.3. Additive Manufacturing and Advanced Lithography for Actuator Fabrication

#### 4.3.1. Additive Manufacturing of Multi-Material

The latest developments in 3D printing allow us to fabricate actuators graded in terms of material properties and functionality. PolyJet printing enables the deposition of rigid and flexible photopolymers simultaneously and produces monolithic actuators with inherent flexures [148]. Functional materials such as conductive inks and piezoelectric composites are directly ink written to provide customized actuator properties. The printing methods that are supported allow for complex internal geometries that were once unfeasible in traditional manufacturing [149].

#### 4.3.2. Sophisticated Lithographic Processes

Two-photon polymerization offers resolution down to sub-diffraction limits to fabricate the actuator features on the nanoscale [150]. In photoresist, grayscale lithography makes it possible to make continuous 3D profiles and to produce smooth actuator surfaces that minimize fluidic resistance. Nanoimprint lithography repeats high-resolution patterns on large scales, which provide the ability to produce a large quantity of complex actuator structures by mass production [151].

#### 4.3.3. Hybrid Fabrication Approaches

The hybrid approach to additive and subtractive methods has been applied to harness the advantages of both methods. 3D-printed templates are used to deposit metal actuators with tailored mechanical properties, using electrodeposition [152]. Complicated internal fluidic channels are formed in monolithic actuator assemblies through printed sacrificial structures. Multi-scale fabrication is a combination of traditionally machined parts and 3D-printed features that are made to work together to deliver maximum performance and cost efficiency [130].

**Figure 16:**
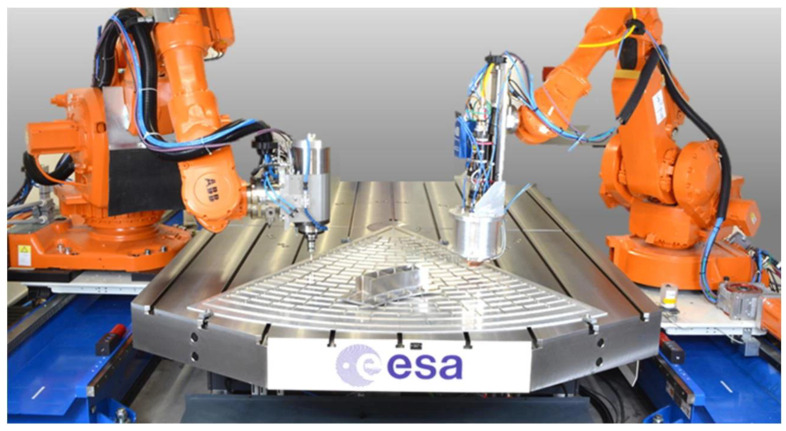
Hybrid manufacturing cell with two robots for laser metal deposition (**right**) and cryogenic milling (**left**) [153].

Figure 16 presents the amazing hybrid technology that is presented by two collaborative robotic arms that collaborate to come up with groundbreaking components [153].

### 4.4. MEMS MEAI-Assisted and Generative Design

#### 4.4.1. Performance Prediction with Machine Learning

Networks are used to predict actuator behavior with deep neural networks that have been trained using finite element simulation data, within milliseconds, hastening the design iteration cycle [154]. Reinforcement learning algorithmic methods are used to optimize control waveforms to complex multi-actuator systems, maximizing performance and minimizing power usage and heat production. Transfer learning allows us to use the knowledge acquired with one type of actuator to design new types of devices [155].

#### 4.4.2. Generative Design Systems

Generative adversarial networks (GANs) generate new actuator geometries with designed performance needs, considering fabrication limits [156]. Compared to human intuition, variational autoencoders are more efficient in their design space and finding non-obvious solutions to multi-objective optimization problems. Physics-informed neural networks make sure that the generated designs will meet the basic physical laws, so the generation process does not require a lot of validation [157].

#### 4.4.3. Automated Design for Manufacturing

The AI systems consider manufacturing constraints directly in the design process, creating geometries designed to optimize individual fabrication processes [158]. Process-conscious design tools can forecast the impact of manufacturing variations to the end device performance, which allows for strong design strategies. The balancing of yield prediction algorithms is between performance maximization and manufacturability to achieve the maximum system reliability and cost-effectiveness [159].

### 4.5. Major Design Trade-Offs and Future Design Paradigm

#### 4.5.1. Management of Performance Trade-Offs

New design approaches clearly discuss the underlying trade-offs between displacement, force, speed and power consumption [160]. The approaches to pareto-optimization are used to find designs that will best balance conflicting goals in particular applications. The adaptive designs adjust their behavior according to operational needs, such that they can give high force when required and high speed when necessary [161].

#### 4.5.2. Reliability Lifetime Reflections

Designing based on reliability involves the use of predictive models of failure modes such as fatigue, stiction and material degradation [162]. Accelerated testing techniques are used to tell the design rules in order to maximize the life of the operation under real conditions. FT architectures also have redundant actuation components and graceful failure modes, and they would continue to operate in the face of partial failures [163].

#### 4.5.3. Future Design Paradigms

The future for MEMS actuator design is to have complete autonomous design systems that keep learning during the fabrication process and performance in the field [8]. Biological designs will use living cells and living tissues as ‘functional components’ to make actuators with self-healing and adaptive properties. Algorithms based on quantum inspiration could be able to solve the design optimization problems that are currently unreachable, which opens up new actuator paradigms [164].

## 5. Multiphysics Modeling Viewpoints and Simulation Structures

Modeling MEMS actuators for application in microfluidics systems requires integrated knowledge of multiple interacting physical domains–mechanical deformation, electrostatic or piezoelectric actuation, heat transfer and microscale fluid flow [165]. The behavior of these systems cannot be explained by just one of the physics disciplines because the deformation of a microstructure directly changes the surrounding electric field and fluid dynamics [166]. Therefore, multiphysics modeling and simulation are essential to accurately predict the performance, reducing the number of design iterations and optimizing the fabrication parameters before physical prototyping. Table 8 outlines some typical multiphysics coupling types in MEMS microfluidic actuators, including those equations that govern these coupling types, the essential physics thematic of the coupling, and what numerical methods are generally used to model and simulate.

### 5.1. Critical Modeling Problems and Technology-Specific Trade-Offs

Simulation MEMS actuators in microfluidic systems does not just require the solution of governing equations; the main difficulty is the development of transferable predictions and experimental consistency of the actuators in more realistic operating conditions. There are three issues of modeling that can be identified throughout the literature. To start with, electrostatic pull-in instability, thermo-fluidic phase change, and piezoelectric hysteresis are strong nonlinearities that most practical microfluidic actuators are governed by in their dynamic behavior. Weak coupling or linearization of the formulation may capture the trends in a qualitative manner, but frequently fail to provide precise predictions of important phenomena such as the stability boundaries, the efficiency metrics, and effects such as long-term drift [178,179]. Indicatively, the actuators can be electrical, and pull-in instabilities are caused by nonlinear electromechanical coupling, making the development of nonlinear model approaches necessary to guarantee the reliability and the long life of the device [178]. Similarly, the piezoelectric actuators require nonlinear constitutive descriptions, capturing hysteresis and energy loss mechanisms for the dynamic response and control accuracy [179]. Second, these actuators change radically in their operation in liquid environments; because it is viscously damped, there are additional mass and changed boundary conditions. Consequently, experiments on models which work in air do not necessarily work in fluidic microchannels. Classical formulations of the hydrodynamic analyses reveal that for viscous fluids, the resonance frequencies and bandwidth and effective damping properties drastically change, requiring fluid–structure interaction (FSI) formulations coupled with multiphysics solvers, which are adequate to reproduce the modified dynamic behaviors [180]. Third, incomplete agreement of the model predictions and experimental measurements have a tendency to persist due to missing physics, including dielectric charging, clamping/stiction effects, material properties dependent on temperature, complexities at the fluid–solid interface, etc. In addition, mechanical and electrical prediction errors in thin-films are also sources of uncertainties that add to the uncertainties in predictions. Consequently, closely knit model calibration and validation structures that entail definite discrepancy functions, and quantification of uncertainty are becoming essential in predictive design and optimization [181]. Technologically, the electrostatic actuators are still appealing owing to their low power consumption and developed electromechanical finite elements modeling (FEM) workflow. Nevertheless, charge dielectric and non-equilibrium surface/contact effects significantly restrict their reliability modeling, which influences the long-term operation [178]. The actuation provided by thermal bubble and electrothermal actuators are bigger in pressure and flow, but necessitate multiphase phase-change modeling which is very resource intensive. Their sensitivity on nucleation assumptions is often the driving factor for the use of reduced-order surrogate models and simplified physics for design iterations [180]. Strong electromechanical coupling with a high response rate, piezoelectric microactuators require complex nonlinear constitutive and fluid–structure interaction modeling in order to capture all effects of hysteresis and losses. Also, the devices have variability, which adds complexity to the portability and generalization of the calibrated models. These observations point out the fact that a more complex model does not necessarily give more useful forecasts unless prevalent unresolved uncertainties and lacking physics are tackled (systematically) in experimental validation schemes [178,179].

Instead of working out the governing equations, this section approaches the subject matter critically to see how various multiphysics couplings are typically modeled in the literature, how they simplify certain physical effects and where the overall predictive limitations lie.

### 5.2. Coupled Physics Domain and Scope of Modeling

The operation of MEMS actuators is inherently multiphysical, with interdependent electromechanical, thermo-fluidic and piezo-electromechanical interactions. Each domain then needs to be modeled and integrated where their simultaneous effects on performance and reliability can be modeled as a coupled system [9,165].

#### 5.2.1. Electromechanical Coupling

**Figure 17:**
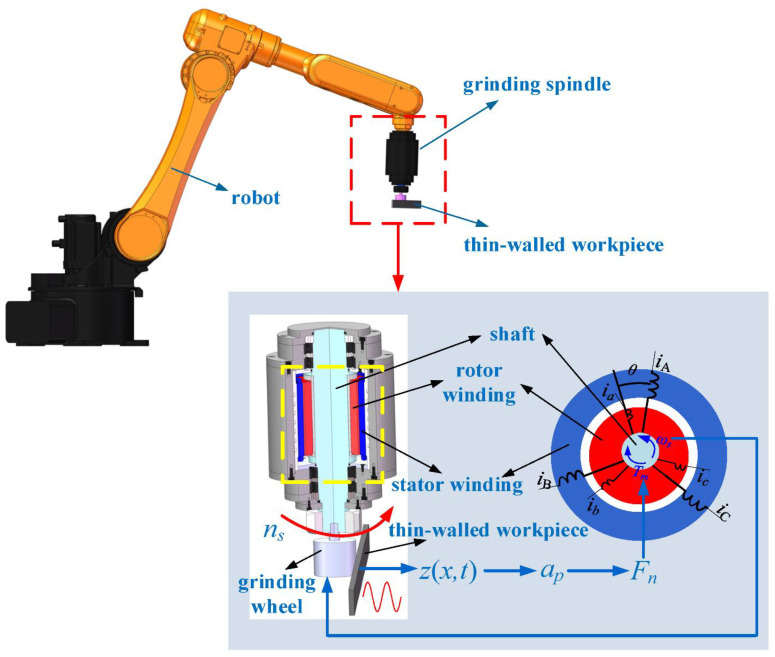
Composition diagram and coupling relationship of robotic grinding system for thin-walled workpiece [183].

Electrostatic MEMS actuators such as parallel plate, comb drive and diaphragm-based actuators are based on an electrostatic attraction between charged electrodes to be converted into mechanical deformation of the movable structure. This basic coupling of the electrical field to the mechanical structure defines the actuator’s performance. For example, the application of a voltage across the electrodes leads to a force which, e.g., pulls the moving electrode towards the fixed electrode and therefore reduces the gap and the capacitance; this leads to a deformation of the electrical boundary conditions and therefore feeds back into the force distribution. The result is a strong nonlinear electromechanical coupling, which must be mathematically described by combining Poisson’s equation for the electrostatics and elasticity or structural-mechanic equations for the mechanical domain [167,182]. As observed in the schematic illustration in Figure 17, the stator is used to convert the input electric energy into a rotating magnetic field and with the interaction between the stator magnetic field and the rotating magnetic field, a driving torque is generated and can be used to move the rotor.

Because of this nonlinearity, phenomena such as the classical “pull-in” instability occur: beyond a critical voltage, the electrostatic force overwhelms the mechanical restoring force and the moving electrode snaps into contact with the fixed electrode [167,184]. In practical modeling frameworks (for example, using commercially available multiphysics tools such as COMSOL Multiphysics or ANSYS Workbench), the coupled system is often solved via iterative methods (for instance, a Newton–Raphson solver), where the mechanical deformation updates the electrode gap and the changed capacitance feeds back into the electrostatic field, and this loop is repeated until convergence is achieved. The necessity of iterative nonlinear solvers is emphasized in the modeling literature [168,182].

The coupling between electrical and mechanical domains also influences resonant frequencies and stiffness characteristics: as the deformation proceeds, the effective stiffness of the movable structure is softened due to the electrostatic spring effect; thus, the resonant frequency shifts. Moreover, structural fatigue and reliability concerns arise because the deformation under high fields subjects the structure to elevated stress and potential dielectric charging or contact fatigue [182].

Finally, advanced actuator designs often must account for the full electromechanical coupling, including fringe fields, residual stress, structural non-idealities, and non-linear dynamic effects such as bifurcations and chaotic responses under harmonic excitation. For example, the work on nonlinear resonances and dynamic pull-in in electrostatic resonators demonstrates how complex the coupling behavior can be [169,185].

#### 5.2.2. Thermo-Fluidic Coupling

In thermal bubble actuators, a microheater embedded in or adjacent to a microchannel rapidly generates localized Joule heating, which raises the temperature of the surrounding fluid and solid substrate to a point where vaporization occurs, forming a transient bubble; this bubble expands violently, displacing fluid and producing a pressure-driven flow that can be harnessed for pumping or valving applications [170]. Capturing this process in a computational model requires solving coupled heat conduction in the solid and liquid domains, phase-change dynamics at the liquid–vapor interface, and Navier–Stokes equations governing the liquid flow, often with interface-tracking methods such as volume-of-fluid (VOF) or phase-field techniques to accurately represent bubble growth and collapse [186].

**Figure 18:**
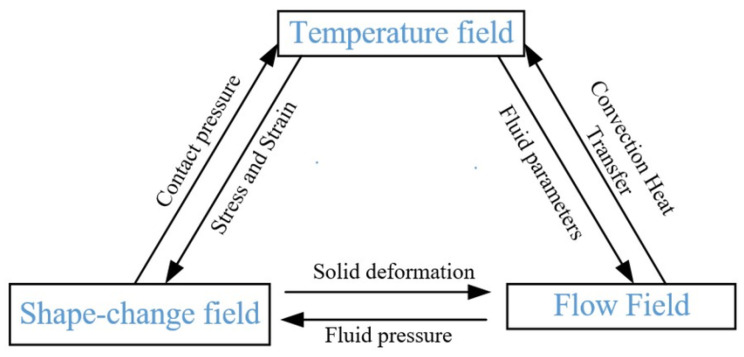
Schematic of thermos–fluid–solid coupling interactions in the wet clutch model [187].

The interaction between thermal and fluid fields is highly nonlinear: the rate of bubble growth depends on the heat flux from the heater and the thermal properties of the substrate, while the expanding bubble induces a convective motion that alters local heat transfer, creating a feedback loop between fluid motion and thermal evolution [171]. The interdependence between the three disciplines is depicted in Figure 18.

Beyond the main thermo-fluidic interactions, there are other secondary effects that can have a significant impact in terms of performance. For example, thermocapillary (Marangoni) flows occur when gradients in surface tension are generated along the liquid–vapor interface, as a result of temperature differences that affect the lateral flows that alter the bubble shape, detachment dynamics, and recirculation within the channel [172]. At the same time, the thermal expansion of the solid substrate as well as the walls of the microchannels changes the channel geometry and affects the site of nucleation and fluid boundary conditions; in some cases, this is even intentionally used to increase the efficiency of actuation [188]. Material properties themselves are temperature-dependent: fluid viscosity, density and surface tension are all temperature-dependent, making it even more difficult to predict flow rates and actuator efficiency.

Because these coupled phenomena occur on very different time scales such as bubble nucleation and growth may occur on a time scale of seconds, while heating of the substrate takes place on a time scale between hundreds to thousands of milliseconds, the simulation must be able to resolve several different spatial and temporal scales at the same time. The conjugate heat transfer between the heater, substrate, and fluid, as well as the latent heat of the phase change, viscous dissipation and pressure transients, thermocapillary flows, and thermal expansion are the performance components that determine the performance of the actuator in terms of flow rate, stroke volume, and efficiency. Therefore, thermo-fluidic coupling represents a central challenge in modeling thermal bubble actuators, requiring fully integrated multiphysics simulation frameworks to capture the dynamic interplay between heat, fluid, and structure in microscale environments [173].

#### 5.2.3. Critical Analysis (Thermal/Bubble Microactuation)

Thermo-fluidic microactuators have the ability to provide relatively high pressure and flow generation, which may be used as a pumping device and a valuing device. However, predictive modeling is highly limited by uncertainties in nucleation criteria, interfacial heat transfer and phase-change dynamics and thus various reasonable choices may produce dramatically different bubble growth histories and net flow predictions. Recent CFD and OpenFOAM-based work points to the fact that the full inclusion of Joule heating and nucleation, as well as phase change, is frequently simplified or otherwise ignored in a bid to make simulations manageable enough to limit the quantitative predictive capability of a simulation, even when qualitative trends are well-captured. Thus, in the case of thermal bubble actuators, the coupling of heat and flow is not the only research gap, but also the creation of experimentally validated reduced-order or hybrid models that can be useful for all operating regimes, without the prohibitive computational cost [189,190].

#### 5.2.4. Piezo–Electromechanical Coupling

**Figure 19:**
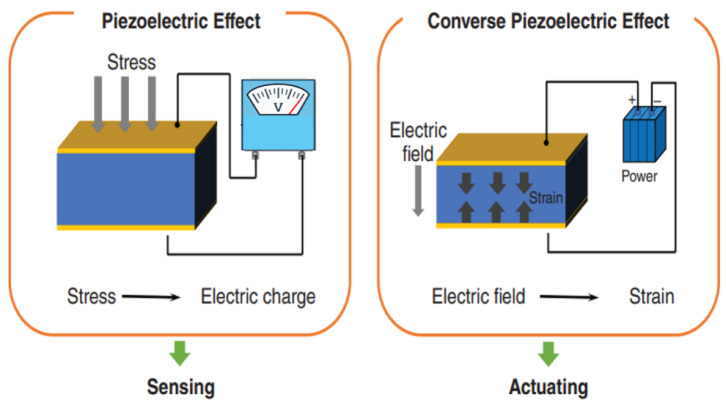
Direct and inverse piezoelectric effects in piezoelectric materials [192].

piezoelectric MEMS actuators, including cantilever-type microvalves and ultrasonic micropumps, exploit the direct piezoelectric effect to convert an applied electric voltage into mechanical strain within the piezoelectric material, thereby inducing motion or pressure in an adjacent fluidic channel [191]. The electromechanical behavior of such devices is governed by constitutive relations that couple the electric displacement field (D) and the mechanical stress field (T) via the piezoelectric tensor, necessitating the simultaneous solution of Gauss’s law for electrostatics and the elastic constitutive equations for solid mechanics [174]. In practical microfluidic implementations, the piezoelectric layer is often bonded to a compliant membrane or cantilever structure that interfaces with the working fluid. This requires fluid–structure interaction (FSI) modeling to accurately capture the transfer of mechanical energy from the vibrating solid into the surrounding fluid, which determines the actuation efficiency, flow rate, and pressure generation within the microchannel [175]. The schematic diagram of the piezoelectric effect in Figure 19 depicts the effect.

The electromechanical–fluid coupling is a nonlinear coupling. Under high electric fields, the piezoelectric layer may show a hysteresis effect, dielectric losses and polarization saturation, all of which will influence the displacement response and the transmitted fluid forces [193]. Furthermore, geometric nonlinearities in the flexible membrane or cantilever can amplify or damp fluid motion, depending on the frequency and amplitude of excitation such that linear approximations cannot predict the actual behavior of the device. In order to achieve a proper simulation, it is necessary to combine the governing equations of piezoelectricity, solid mechanics and incompressible fluid flow in an integrated manner, typically using finite element or multiphysics numerical methods. Such fully coupled multiphysics models are necessary to predict dynamic effects such as transient flow generation, resonance behavior, energy dissipation, and nonlinear interaction effects that show that decoupled single-physics analyses are not able to reflect the real operation characteristics of MEMS microfluidic actuators. Such interdependency of mechanisms adds to the complexity and calls for stringent computational modeling of piezoelectric MEMS devices in order to design, optimize, and control them [174,175,193].

#### 5.2.5. Critical Assessment (Piezoelectric Microfluidics)

Piezoelectric actuators are precise and provide excellent electromechanical coupling, and fully coupled FSI models are able to model diaphragm–fluid energy transfer in a more realistic manner compared to decoupled workflows. Nevertheless, a notable weakness is that linear piezoelectric constitutive equations can be excessively predicted to indicate displacement and pressure response in the presence of hysteresis, dielectric loss, field-dependent nonlinearity, and so on, at realistic drive frequencies. Moreover, batches of thin-film piezoelectric devices may be heterogeneous, and this is why the portability of a calibrated model is lowered, thereby encouraging parameter identification and uncertainty quantification as a modeling task. Consequently, the way to fill the greatest gap in piezoelectric multiphysics modeling is not to do so, but to obtain device-independent predictive models that will be maintained under different drive conditions and aging behavior [194,195].

### 5.3. Techniques of Multiphysics Simulation: Numerical

As opposed to giving tutorial-like descriptions of numerical methods, this section critically accounts how the techniques are used in the literature in relation to MEMS microfluidic actuators, and where their key predictive advantages and constraints occur.

An explanation of multiphysics behavior is only possible through advanced numerical techniques that are capable of dealing with nonlinearity, transitions, and complex boundary interactions at the microscale.

#### 5.3.1. Finite Element Modeling (FEM)

FEM is still the pillar supporting the MEMS actuator simulation, due to its potential for discretizing the complex geometries and solving coupled partial–differential equations over the irregular domains. In the FEM framework, the physical domain is divided into small finite elements, within which field variables (e.g., displacement, potential, temperature) are approximated via shape functions, and the global system of equations is assembled and solved iteratively [167]. In the context of MEMS design, FEM is extensively used to simulate mechanical deformation (e.g., stress/strain in beams, membranes), electrostatic field distributions (e.g., potential and charge around electrodes), and thermal stresses (e.g., due to Joule heating or thermal expansion) with high spatial fidelity. For example, FEM has been applied to model electrostatically actuated microsystems, including fringing-field effects to improve accuracy [176,196].

Commercial multiphysics platforms such as COMSOL Multiphysics and ANSYS Mechanical provide ready-to-use modules for coupled electromechanical, thermal–structural, and fatigue analyses within a unified computational domain. Studies on electrothermal MEMS actuators demonstrate the need for coupling between heat conduction in solids, thermal expansion and structural deformation, which FEM handles efficiently [197].

Another critical capability is parametric sweeps: designers can vary key parameters such as electrode spacing, material thickness, actuation voltage or membrane geometry, and examine how these influence the overall displacement, resonance frequency, response time and stress distribution. For instance, analytical and FEM modeling of thermal flexure actuators in COMSOL showed how voltage and geometry variation change the deflection and temperature distribution [198,199].

Although they have been widely used, FEM-based multiphysics models frequently assume idealized boundary conditions and idealized bulk material behavior, which restricts their ability to be applied on a quantitative basis to predict thin-film MEMS actuators in liquid environments. As a result, most of the published FEM works are qualitative trends that need to be calibrated by a lot of experimentation to be reliable.

#### 5.3.2. Computational Fluid Dynamics (CFD)

CFD techniques are applied to model the fluid flow and pressure variations generated by actuator motion in micro-scale fluidic systems. At the microscale, the Reynolds number is typically very low, leading to laminar flow regimes; nevertheless, accurate modeling of velocity, pressure and shear stress is essential. CFD simulations typically solve the incompressible (or weakly compressible) Navier–Stokes equations, along with the continuity equation, and impose appropriate boundary conditions such as no-slip (for continuum regimes) or slip (when the Knudsen number indicates rarefaction) at the walls. The importance of properly treating slip–flow regimes at micro-scales has been documented [200,201].

CFD supports the prediction of essential performance parameters such as volumetric flow rate, pressure gradients, shear stresses acting on delicate samples (e.g., in biomedical microfluidics) and flow-domain responses to moving boundaries. When the actuator surface deforms, coupling the CFD solver with moving mesh or arbitrary Lagrangian–Eulerian (ALE) methods enables accurate tracking of the fluid domain’s evolution and the solid–fluid interface motion regimes [202,203].

Although CFD allows for detailed visualization of flow fields at the microscale and offers strong localized position estimation, regardless of whether these edges are mobility walls or flowing walls, traditions, methods or approximations that characterize its approach to the field including the assumed constant fluid behavior between surfaces as Newtonian, no-slip boundary conditions, and moving-boundary simplifications are highly limiting to both high-frequency and strongly coupled actuation.

#### 5.3.3. Multiphysics Solvers and Co-Simulation Frameworks

Modern simulation practice for MEMS actuators employs multiphysics solvers and co-simulation frameworks, where FEM and CFD solvers are integrated to resolve multiple coupled domains electrical, thermal, structural and fluidic within a consistent computational environment. For example, a micropump based on a piezoelectric diaphragm can be simulated by using FEM to compute diaphragm deflection (structural/piezoelectric domain) and CFD to analyze fluid displacement (fluidic domain), with a boundary-data exchange at each time step until convergence. Commercial software such as COMSOL Multiphysics and ANSYS Multiphysics and open-source options like OpenFOAM (coupled with structural solvers) support such direct matrix coupling or partitioned time-stepping schemes. Many recent studies highlight the significance of strong coupling between domains: for example, multiphysics analyses of electrothermal actuators integrating finite-element thermal/electrical models with surrounding fluid convection via finite-volume methods [197,204].

These platforms further support advanced techniques such as adaptive meshing (refining mesh in regions of high gradients), time-stepping control (for resolving fast transient phenomena such as pull-in or collapse), and moving-mesh or ALE methods to handle deformable boundaries under large nonlinearities [180].

Fully coupled solvers are more physically faithful, but as they are often of high computational cost and are sensitive to poorly known parameters, they may only be used in case studies and not in general design rules.

### 5.4. Model Calibration and Validation Using Experimental Data

The integrity of a multiphysics simulation ultimately depends on its ability to reproduce experimentally observed behavior. Additionally, the concept of calibration and validation is articulated as the core of predictive modeling for MEMS actuator research, which links the gap between numerical abstraction and physical realization. According to Ling et al. [205], compared to simulation results with experimental data, the calibration of multi-physics computational models using Bayesian networks process enables us to reduce uncertainties and enhance the confidence and reliability of predictive models when the system is analyzed from a coupled electro–thermo–mechanical system.

Calibration is the first step in this process, which is the systematic adjustment of poorly known physical constants (e.g., Young’s modulus, residual stress, damping coefficients, piezoelectric constants) until the result obtained by the simulation agrees with the measured data. Experimental calibration of micro electromechanical relays using laser-Doppler vibrometry (LDV) has shown that dynamic deflection data can be used to obtain reliable material stiffness and damping properties, resulting in optimized finite element models for the prediction of displacement and stress evolution with high fidelity [206].

For accurate model parameterization, multiple experimental instruments with the ability to capture microscale mechanical and fluidic effects are utilized. LDV is a non-contact vibration and deflection measurement system with nanometer confidence, which is suitable for the characterization and evaluation of the resonant frequency and mode shape of piezoelectric and electrostatic actuators. White light interferometry is capable of capturing static out-of-plane deformation and residual stress gradients, whereas micro-particle image velocimetry (μ-PIV) is used to record transient flow fields and pressure distributions through microchannels. For instance, experimental validation of the computational fluid dynamics model using micro-particle image velocimetry showed that micro-PIV data could successfully measure local velocity profiles, which were able to validate simulated flow regimes in micro-pumping systems [207].

Model validation is not limited to calibration; numerical predictions must be valid far outside of the calibration regime in terms of the operating conditions and experimental boundary conditions. This is typically done by a direct comparison between simulated and experimental curves, such as deflection–voltage curves for electrostatic actuators or flow–pressure curves for thermal micropumps. An example includes the application of LDV-based dynamic characterization in determining the validity of frequency response models under changing environmental conditions, which is provided [208].

However, even if the calibration is conducted rigorously, due to unmodeled effects such as dielectric charging, leakage via micro-scale pathways or surface adhesion, discrepancies often arise. These discrepancies are useful hints of missing physics or approximations in the computational model. In particular, the Bayesian calibration formalism proposed by Kennedy and O’Hagan defined a model discrepancy function to capture the systematic differences between simulation and experiment to improve their iteration refinement and uncertainty quantification [205].

To overcome these long-standing model–experiment mismatches, inverse modeling techniques are currently being used, in which experimental observations are iteratively used to update experimental inputs for the model, such as parameters and constitutive relations. Such hybrid techniques have been very effective in MEMS device characterization, which combined machine learning algorithms and experimental data to reconstruct accurate electromechanical parameters and enhance the design reliability. With these integrated calibration and validation workflows, multiphysics models transform from being theoretical abstractions to quantitative predictive models that can be used to inform next-generation MEMS actuator design [209,210,211].

### 5.5. Limitations in Simulation Accuracy and Computational Cost

In spite of superb progress, there are still a number of downsides, limiting how realistic and scalable MEMS multiphysics modeling is.

Computational cost: Transient fully coupled 3D simulations are very power-intensive and require large memories. The high-fidelity models that incorporate fine-meshing of the microchannels, electrodes, and moving interfaces usually lead to a simulation time of several days.

Material uncertainty: The mechanical and thermal properties of thin films such as silicon nitride, aluminum nitride, or SU-8 differ from their bulk counterparts, due to the grain boundary and surface effects. Inaccurate parameterization of these properties leads to deviations between simulated and experimental results.

Boundary condition simplification: Many simulations assume ideal boundary conditions (perfect insulation, no-slip walls, or fixed supports). However, real-world MEMS devices experience imperfect clamping, stiction, and temperature gradients that are difficult to model precisely [212].

Neglect of multiscale effects: Processes such as the fringing of electric fields, flow of rarefied gases, and loss of surface energy happen at the nanometer scale and cannot be modeled using the continuum. To include these effects, it is necessary to couple a molecular dynamics (MD) or lattice Boltzmann methods (LBM) solution to the continuum solvers, which is a computationally intense method [213].

Fluid–structure interaction approximation: Linearized FSI models fail to capture nonlinear fluid responses, especially in oscillatory or high-frequency actuation. This limits the predictive capacity of models for applications like acoustic streaming and ultrasonic pumping [214].

Addressing these challenges calls for reduced-order models (ROMs) and data-driven surrogate models that retain essential multiphysics fidelity while significantly lowering the computational costs.

## 6. Performance Optimization and Reliability Enhancement

### 6.1. Optimization Metrics

Microfluidic MEMS actuators must be co-designed around the four metrics that rarely move independently. The actuation force sets the maximum back-pressure that a pump or valve can overcome and governs the sealing margins at the seat. Piezo-driven diaphragms and stacks offer high force density, whereas electrothermal beams trade force for heat and power unless the geometry and pulse shaping are tuned to confine temperature rise. Piezo pumps and electrothermal actuators detail these modality trade-offs and typical operating envelopes [215].

#### 6.1.1. Actuation Force

Actuation force defines the maximum back-pressure a microvalve or micropump can overcome and directly determines the sealing performance, pressure head, and throughput [216]. In piezoelectric systems, the force density can exceed 10 MPa due to the intrinsic coupling between the electric field and strain in materials, while the same structures can operate in both direct and reverse actuation modes [217]. By comparison, the electrothermal actuators like V-shaped or chevron beam arrays produce large displacements and reduced forces, since they rely on thermal expansion instead of inherent electromechanical coupling [218]. The nature of the trade-off is that higher temperatures used to get even more expansion place thermal stress and material fatigue upon the actuator. New hybrid technologies such as electro-thermo-piezo composites and metal–polymer multi-layers have been suggested with an attempt to enlist a balanced high force output with better thermal and reliability characteristics [219].

#### 6.1.2. Displacement (Stroke)

Mechanical stroke of a valve membrane or pumping diaphragm is controlled by displacement and the amount of fluid volume displaced per actuation cycle. As the displacement is increased in general, it enhances the throughput at the expense of creating large membrane stresses, decreasing the resonant frequency and nonlinear effects in stiffness. In response to this, topologies have been developed to enhance the stroke whilst not degrading the mechanical integrity, by effective topologies like corrugated membranes and bimorph diaphragms [174,220]. It has been demonstrated in fluid–structure interaction (FSI) modeling that parameters like the chamber aspect ratio, inlet/outlet geometry and membrane thickness prevail in the stroke–flow interaction. The response surface methodology or design of experiments is now frequently used as an optimization strategy to discover these dominant factors prior to complete genetic or gradient-based optimization [221]. In addition to this, through finite element modeling combined with computational fluid dynamics (FEM CFD) it is possible to predict nonlinear deformation and pressure–velocity distribution simultaneously, and this prediction of the displacement is better-predicted [177,180].

#### 6.1.3. Response Time

The most important indicator of the dynamic performance is the response time; this is a factor that is dependent on the mechanical inertia of the moving components and the energy transport mechanism. In the case of piezoelectric actuators, resonant frequency and structural damping control switching speed, it is possible to create frequencies at speeds of and above 10 kHz with thin-film PZT membranes [222]. The heating/cooling rate in electrothermal actuators is characterized by a thermal RC constant

*τ* = *ρcV/G*

where *ρcV* is the heat capacity and *G* is the thermal conductance [223]. By reducing the length of heat-paths, low-thermal-mass materials (e.g., SU-8, polyimide), micro-heat-sinks or thermoelectric cooling may go a long way to shorten response times [107,224]. The piezoelectric resonance frequency has demonstrated a high actuation speed, maximized actuation speed with reduced overshoot and hysteresis using advanced pulse-width modulation (PWM) and waveform shaping. Comparison between vacuum and ambient control also demonstrates that squeeze-film damping, along with convective losses, have a significant impact on transient dynamics where packages and environment are important design parameters [225].

#### 6.1.4. Energy Efficiency

Energy efficiency represents a system-level property that captures not only actuator power consumption but also total work delivered per cycle, losses due to viscous drag, leakage, and mechanical hysteresis. In valveless diffuser nozzle piezo micropumps, geometric optimization of the diffuser angle and chamber volume has improved the flow-rate-per-watt [181]. Thermal actuators, on the other hand, are inherently less efficient because much of the input energy dissipates as heat. Emerging materials such as low-loss piezoelectrics (Sc-doped AlN), electrostrictive polymers (PVDF-TrFE), and shape-memory alloys (NiTi) are being adopted to enhance the mechanical-to-electrical conversion ratio [124]. The system-level design also considers driver electronics and waveform control, using impedance matching, resonant driving, and energy recovery circuits to further improve efficiency [226,227]. Table 9 summarizes the important performance metrics, prevailing design factors and modeling procedures related to the optimization and reliability of MEMS microfluidic actuators.

### 6.2. Multi-Objective Optimization Methods

**Figure 20:**
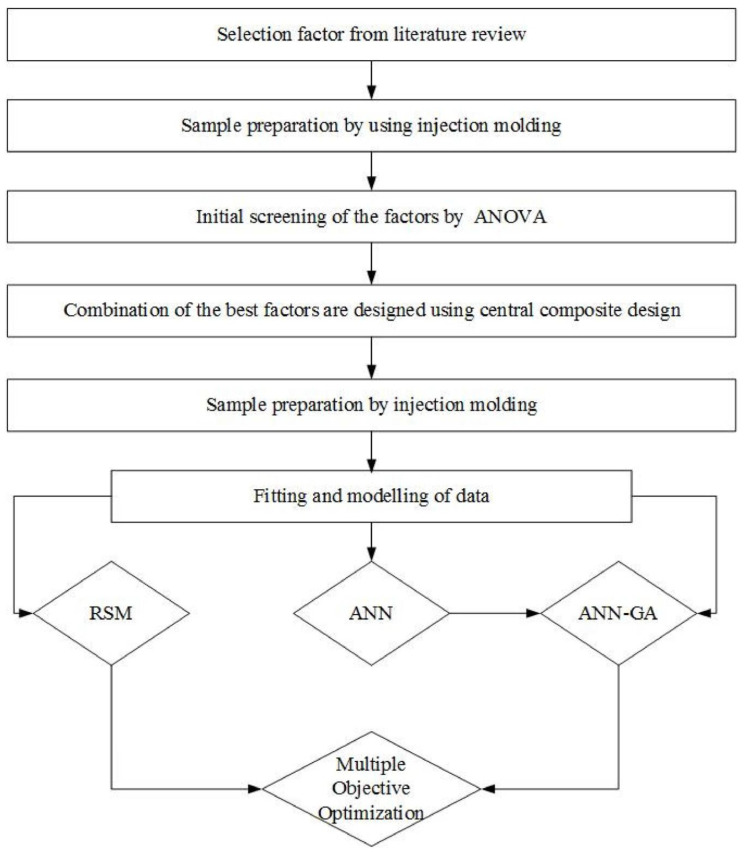
Flow diagram indicating the order of experimental design and the modeling techniques [229].

Multi-objective optimization methods comprise the design of experiments (DOE), response surface methodology (RSM), genetic algorithms (GAs) and machine learning (ML). Figure 20 illustrates the sequential framework adopted for integrating an experimental design with modeling approaches.

#### 6.2.1. Design of Experiments (DOE)

DOE is a systematic statistical approach used to plan and analyze experiments efficiently by varying multiple design factors simultaneously to determine their influence upon system performance [230].

In microfluidic actuator design, DOE helps identify key geometric, material, and operational parameters such as membrane thickness, channel width, or voltage amplitude that significantly affect outputs like actuation force, displacement, or flow rate [208]. The method works by generating structured experimental layouts such as full factorial, fractional factorial, or Taguchi designs that reduce the number of required trials while maintaining statistical significance.

DOE will allow researchers to identify the primary effects, interaction effects, and nonlinear trends between factors and offer insights that can be used in the further optimization of the factors, using techniques such as RSM or genetic algorithms. The strong points are that it is highly efficient in data collection, the cost of the experiment can be lowered, and the possibility of quantitative identification of the influential variables, which make it a desirable initial step in multi-objective optimization. Nevertheless, the DOE cannot be used with complex, very high-dimensional, or strongly nonlinear systems, because it relies on smooth response surfaces and may become computationally limited and less accurate with large factor space. Nevertheless, the DOE is still being considered as a pillar in the scheme of experiment design in microfluidics and actuator performance improvement [231].

#### 6.2.2. Response Surface Methodology (RSM)

The RSM is a mathematical and statistical methodology that is employed to model and analyze relationships between one or several input variables and one or several response outputs. The RSM is generally used with the DOE to create a surrogate model (usually second-order poly) to approximate the surface of the system response in the microfluidic actuator design [226,232]. The procedure allows for visualization of the influence of design parameters, including the geometry of the actuator, the rigidity of the material and voltage on performance measures, including displacement or energy consumption [233]. The RSM helps to solve the problem of the size and shape of the optimal design without undertaking a great number of experiments, both physically and through simulation. The RSM’s strengths encompass that it may manage multiple factors effectively, produce an interpretable mathematical representation and facilitate sensitivity and optimization analysis. However, it has disadvantages where the system response is very nonlinear or discontinuous, because the low-order polygonal approximations in the focus might not portray its complicated behaviors. Nevertheless, the RSM is still a popular and affordable device of early-stage actuator optimization [234,235].

#### 6.2.3. Genetic Algorithms (GAs)

GAs are population stochastic optimization techniques based on the action of natural selection and genetics. Multi-objective actuator design GAs can be used to evolve a population of candidate designs by applying iterative selection and crossover and mutation to select a Pareto-optimal trade-off between conflicting design objectives (force, speed, and energy consumption). One of these algorithms is the NSGA-II algorithm, which is especially beneficial for searching against large, nonlinear, and multi-modal design spaces, which are common with soft and microfluidic actuators [236].

A multi-objective GA made a stroke actuator (pneumatic) 30 percent more efficient with less displacement at full power loss [237]. The advantages of GAs are that they have the ability to search over a global scale, hardiness to local minima, and elasticity to coping with more elaborate objective functions. Their weaknesses, however, are relatively high costs of computation, and the fact that they require the optimization of parameters (population size, mutation rate), which may have an effect on the quality and speed of convergence [238].

#### 6.2.4. Machine Learning (ML)

ML is finding more applications in microfluidic and soft actuator optimization, where data-driven models can be built to predict performance results and help us to quickly explore designs. Far more complicated nonlinear relationships between the design variables (geometry, pressure, voltage) and actuator responses (displacement, flow rate, or energy consumption) can be taught with no conscious physical modeling by using ML algorithms, which include neural networks, support vector regression algorithms, and Gaussian processes [239]. The latest applications have combined Bayesian optimization and physics-informed neural networks (PINNs) to actuator simulation to find the optimal design with a much smaller number of evaluations. The contributions to the strengths of ML are that it is capable of working with high-dimensional data, has nonlinear dependency, and it can also save on optimization time, since it provides alternative simulation-based cost-prohibitive CFD and FSI. But ML models are data-contained and can frequently need big and illustrative datasets, and they lack physical interpretability without being constrained with physics. Nevertheless, by using ML-driven models, the optimization of actuators in the future is changing to be a rapid, adaptive and autonomous design process [239,240].

### 6.3. Reliability Issues

Microfluidic actuators confront a diverse spectrum of reliability threats, many of which parallel those identified in MEMS and soft-device domains. For instance, thermal stress and fatigue arise from differential thermal expansion during fabrication or actuation, localized heating in power components, and cyclic thermal loading, leading to creep, delamination or fatigue cracks in thin films and elastomeric membranes [241]. Next, mechanical fatigue and wear result from repeated membrane deformation, moving seals or parts, and when particulate-laden fluids are used, erosion accelerates surface degradation, crack initiation and loss of sealing [242]. In addition, soft-polymer materials such as Polydimethylsiloxane (PDMS) can undergo material degradation and swelling, absorbing solvents or undergoing oxidation or hydrolysis, which alters their mechanical properties, permeability and geometry over time [243]. Further, stiction, fouling and bio-contamination present critical challenges in biosystem applications; capillary forces, surface energy heterogeneities, protein adsorption and biofilms can cause moving parts to stick or channels to block, altering wetting and flow behavior [244]. Finally, manufacturing variability and assembly-induced defects such as layer misalignment, bonding defects and residual stresses lead to device-to-device variability, decreased yield and unreliable lifetime prediction. Taken together, these failure mechanisms underscore the imperative for reliability-driven design strategies, accelerated life-cycle testing, and careful material and process selection in microfluidic actuator systems [245,246].

### 6.4. Strategies for Robust Design and Long-Term Performance Stability

The optimization of materials, stress-sensitive design of microfluidic actuators, protective packaging and data-driven reliability management must be combined to ensure the strong performance and long-term stability of microfluidic actuators. Material selection and surface modification is one of the most basic strategies. By selecting polymers and elastomers that have minimal solvent absorption, high fatigue levels and thermal stability like the fluorinated elastomers or polyurethane composites, swelling and chemical degradation can be drastically cut down through time. In addition, anti-fouling surfaces and oxygen plasma treatments have proved to reduce protein adsorption, stiction and biofouling in microchannels and thus enhance the reusability and longevity of devices [132,247]. Geometry- and stress-conscious design is another big consideration. The finite element and fluid–structure interaction (FSI) simulation permits the designers to see the distribution of stresses, pinpoint areas of weakness, and ratios of thinness to span, radius, and curvature to mold a joint so that the local stress concentrations are minimized. Research indicates that integrative compliance flexures and rounded transitions alleviate stationary wear and tear, as well as increase the durability of the actuator cycles [248]. Encapsulation and packaging strategies help in enhancing environmental and chemical robustness. Multilayer barriers, parylene and PDMS–glass hybrid encapsulation deters moisture and solvent adsorption to preserve mechanical and electrical parts because of hermetic sealing. Such approaches are particularly essential in the case of implantable and wearable bio-micro devices that have to work in fluid-rich environments [66,133].

Finally, process control and quality assurance (QC) are essential for minimizing manufacturing variability. Inline metrology, statistical process control (SPC), and post-fabrication inspection frameworks (e.g., optical profilometry and leak testing) have been incorporated to improve consistency and yield in microfabricated actuators [249,250].

## 7. Applications and Case Studies

MEMS-based actuators are a key component in the development of modern microfluidic systems by providing a means of programmable, precise and scalable fluid and biological sample manipulation that occur by laminar flow. Active actuation processes are vital to overcome diffusion limited transport, accomplish deterministic droplet operations and control mechanical stimuli and microscale environments that are pertinent for biological retrieve and lab-on-chip integration [63,251]. Accordingly, applications of MEMS-enabled microfluidics are most meaningfully discussed in terms of actuator-centered case studies that put considerable emphasis on the explicit connection of biological sample constraints to actuation choices, rather than purely descriptive end-use.

**Figure 21:**
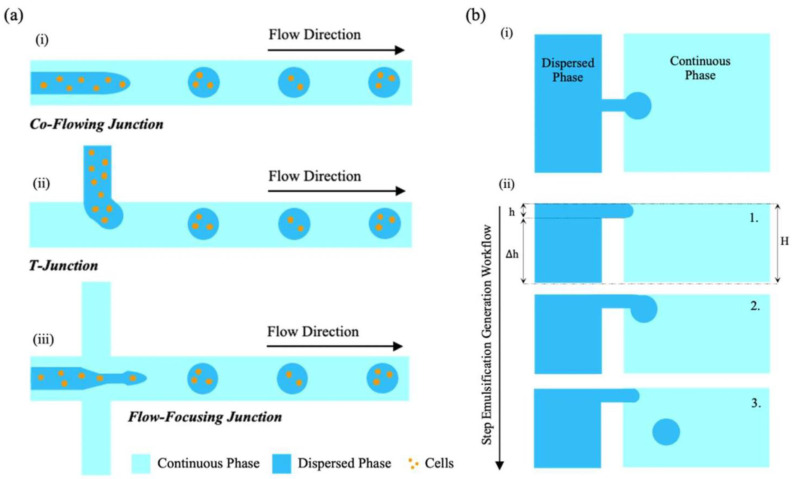
(**a**) Schematics of droplet-based microfluidic strategies for the generation of cell-laden hydrogel microcapsules, illustrating three commonly used device architectures: (**i**) coaxial flow, (**ii**) T-junction, and (**iii**) flow-focusing junction. (**b**) Schematic representation of the step emulsification mechanism: (**i**) top view of the microfluidic architecture, showing the dispersed phase flowing through a shallow nozzle into a deep reservoir filled with the continuous phase, and (**ii**) side view, illustrating the cross-sectional sequence of droplet formation. These droplet operations rely on precisely controlled flow rates and interfacial dynamics, which are enabled and stabilized by MEMS-compatible actuation (e.g., pressure-driven flow control, pneumatic microvalves, or electrohydrodynamic modulation), allowing deterministic droplet generation and encapsulation for biological and bioengineering applications [251].

The practical applications of microfluidic and MEMS-based technologies expanded in biological and biomedical systems. Figure 21 illustrates droplet-based microfluidic strategies for cell encapsulation and step emulsification, demonstrating how controlled microscale architectures enable precise cell handling, compartmentalization, and functional bioengineering applications.

### 7.1. Droplet Generation, Encapsulation and Encounter

Droplet-based MFC is commonly utilized for compartmentalized biological assays, single cell analysis and co-cultures. Active MEMS actuators allow one to obtain deterministic droplet formation, transport, splitting and merging, which can hardly be achieved by passive breakup mechanisms only. Some of the most commonly used methods to control droplet size, timing and routing with high reproducibility include electrowetting on dielectric (EWOD), pneumatic microvalves, and piezoelectric actuation [252,253]. Droplet encapsulation has been widely used for applications in studies of cell culture and cell–cell interaction, in which droplets can be employed as isolated microreactors to maintain biochemical microenvironments and allow massive parallelization. Valve- or EWOD-controlled droplet systems also enable selective droplet fusion, reagent addition, and being time-dependent, they can be used for multi-step workflow, stimulation-response assays, cell–cell interaction, and drug-screening platforms [254,255].

### 7.2. Microfluid Mixing, Pumping and Flow Control

At the microscale, the flow is predominantly laminar, while diffusion-limited transport limits the mixing effects. Actuators based on MEMS overpower these limitations in the sense that they actively perturb the flow field. Electrothermal actuation creates localized temperature gradients that produce thermocapillary convection, and piezoelectric actuation creates acoustic streaming that can enhance mass transport using no complex channel geometries or external pumping structures [251]. For the transport of fluids, MEMS micropumps, on the basis of piezoelectric, electrostatic or thermopneumatic principles, allow for accurate dosing and recirculation in the nL–µL·min^−1^ range. Piezoelectric micropumps demonstrate high resolution flow rates and a high response time, whilst a thermopneumatic approach afforded higher stroke volumes at lower frequencies so as to provide sustained perfusion rates and reagent delivery in biological assays [256,257].

### 7.3. Manipulation of Cells/Cell Particles

MEMS actuators are widely applied to the manipulation of cells and microparticles with minimal mechanical attrition. The contactless handling/sorting/focusing is possible with acoustic and magnetic actuation, which is especially beneficial for delicate or label-free biological samples. Surface acoustic wave (SAW)-based devices, for instance, have shown high-resolution manipulation of single cells with high viability and functional integrity [258]. Magnetic MEMS-based methods further enable selective manipulation of magnetically tagged cells or particles, which supports applications such as immunoassays, rare-cell isolation, and separating desired cells of interest, among others, in heterogeneous biological samples. These actuation strategies are normally combined upstream from analytical modules in terms of sample preparation and enrichment [259].

### 7.4. Integration in Lab-on-Chip Systems and Organ-on-Chip Systems

In LoC platforms, MEMS actuators are used to automate diverse and intricate biochemical flow charts, such as the metering of samples, the introduction of reagents, and the mixing, incubating, and removal of wastes. Such automation allows high-throughput testing, multiplexing and less reagent usage, with the advantages of better repeatability and robustness of integrated biosensing systems [260,261]. Organ-on-chip (OoC) systems are based on the usage of MEMS actuators to recreate physiologically relevant microenvironments. Pneumatic and piezoelectric actuators distort flexible membranes to produce controlled shear stress, pulsatile flow and cyclic strain, enabling the recreation of physiological models of cardiac, pulmonary and vascular tissues to screen drug responses and model disease [262,263].

### 7.5. Biological Sample Constraints → Selected Actuation

While MEMS actuators can allow for a wide range of types of operation for microfluidic operations, the selection of the actuator comes with severe limitations, based upon the nature and sensitivity of the biological matter in question. A framework to guide decisions that is consistent with sample class, dominant constraints and actuator trade-offs is, therefore, critical to rational microfluidic system production [255].

#### 7.5.1. Biological Sample Classes and Preponderant Sensitivities

Motile microswimmers (e.g., sperm, motile bacteria) are very sensitive to the hydrodynamic shear stress and surface interactions. Channel geometry, curvature and properties of walls have substantial effects on motility, trajectory and transport efficiency in favor of smooth-flow, low-shear actuation strategies [264]. Mammalian cell response to stress in the form of shear stress and temperature elevation is very sensitive and can impair membrane integrity, gene expression and long-term viability. Actuation strategies for mammalian cells must therefore have low mechanical stress and low thermal loading, especially in long-term culture/stimulation experiments [258,265]. Organoids and embryos necessitate tightly controlled microenvironments, which are characterized by low shear stirring, continuous perfusion, high sterility and mechanically stable environments for long periods of time. These requirements favor actuator architectures delivering smooth, low frequency control of flow and with low mechanical fatigue, e.g., pneumatic or piezoelectric membrane actuation [262,266]. Extracellular vesicles and proteins/enzymes are mainly limited by adsorption, surface fouling and chemical compatibility and not so much by mechanical stress. Material choices, surface chemistry and reduction in interfacial contact dominate the choice of actuator and microfluidic design from these samples [67,255].

#### 7.5.2. Actuator Specific Risks and Advantages

Electrothermal actuators are a powerful actuation source, but will cause a local heating of Joule heating that may adversely affect temperature sensitive biological samples. EWOD-based actuation can be used to control fluids in the form of droplets very precisely, but is susceptible to biofouling and protein adsorption at electrode surfaces [248,249]. Pneumatic microvalves provide gentle and biocompatible control for prolonged culture, but though membrane fatigue and leakage, can be rate-limiting for durability. Piezoelectric actuators allow for easy, low-shear actuation with a low temperature rise and with the cost of fabrication and driving voltage requirements [123,267].

#### 7.5.3. Examples of Representative Case Studies

Motile microswimmer case: Studies of sperm transport show that the channel curvature and surface interactions are powerful factors in the motility and navigation of the spermatozoa, and are all conducive to low-shear, smoothly actuated environments in microfluidics [264].

Long-term cell and organoid culture: High-throughput three-dimensional cell and organoid culture platforms employ pneumatic or piezoelectric actuation to enable stable perfusion and mechanical signals over long periods of time, with minimum emphasis on the relatively long lifespan, considering reliability, sterility and low mechanical fatigue [262,266].

Droplet-based cell culture/interaction: Droplet microfluidics allow for compartmentalized culture and the interaction of cells in controlled microenvironments, where actuator-driven droplet manipulation allows multi-step biological workflows of programmable fusion and splitting, as well as reagent exchange [255].

Together, these examples illustrate how direct knowledge of sample-specific constraints helps to provide information about the suitable selection of actuators and architectures and arcs, and how it transforms applications from describing their behavior as a demonstration to showing those that serve as rational decision-guiding designs.

### 7.6. Comparative Assessment and Commercialization Issues

Across applications, the choice of actuator involves imposing and compromising force, displacement, response time, power consumption, complexity of fabrication and long-term reliability [63,268]. Hybrid actuation strategies utilizing complementary mechanisms are becoming a focus of more research in order to balance between these trade-offs for a variety of different microfluidic tasks. From the point of view of commercialization, the focus is changing towards reproducibility, biocompatibility and regulatory compliance, which are essential for the translation of MEMS-enabled microfluidic systems from demonstration in the laboratory to deployable biomedical tools [113,269].

## 8. Challenges, Opportunities, and Future Perspectives

Although MEMS actuators have made great strides in microfluidic applications, there are still a number of challenges that cannot be resolved yet and a number of new opportunities are being realized. This part discusses the existing constraints, the potential avenues of research and the future of this fast-growing discipline [11].

### 8.1. Design and Fabrication Challenges at Sub-Micron Scales

#### 8.1.1. Scaling Limitations and Quantum Effects

The traditional scaling laws start to break as MEMS actuators reduce to sub-micron sizes, and quantum mechanical effects start to play a more important role [89]. Volumetric forces are overshadowed by surface forces, such as van der Waals interactions, electrostatic forces, and capillary effects, and cause stiction effects and unstable devices. Significant nanoscale features of sufficient precision and scalability have not been made so far, especially in materials with multi-material structures. The smaller the scale, the harder thermal management is, because the heat capacity is lower and the power density is higher [270].

#### 8.1.2. Material Limitations and Interface Engineering

The existing MEMS materials show deterioration of their performance at the micro and nano scales, and the thin films also have different characteristics of the bulk materials [105]. The interface engineering issues are adhesion failure, stress-induced delamination, and diffusion-based degradation. The creation of new nanomaterials such as 2D materials, metamaterials, and nanocomposites has been found to have opportunities as well as fabrication challenges. The issue of biocompatibility increases further as the dimensions of the devices get closer to the cellular and molecular dimensions [96].

#### 8.1.3. Fabrication Precision and Yield Challenges

A high feature control of less than 10 nm feature with a high yield is still costly to many MEMS applications [271]. Multi-material integration has a problem with thermal expansion mismatches, chemical incompatibilities and limitations with the process sequence. Three-dimensional nanofabrication methods such as the two-photon polymerization and focused ion beam milling have a high resolution with a low throughput and expensive nature. The measurement and the characterization of a structure at the scale of nanotechnologies is a challenging task in regard to quality control and performance verification [2,11,57].

### 8.2. Modeling Gaps in Nonlinear Dynamics and Coupled-Field Interactions

#### 8.2.1. Multiphysics Coupling Issues

The existing modeling tools frequently simplify interactions among various physical domains, thus causing poor performance predictions [9]. Fluid–structure interaction frameworks are not good at explaining micro-scale phenomena such as slip boundary conditions, non-Newtonian dynamics and dominance of surface tension. Depending on the electro–thermal–mechanical coupling, nonlinearities are introduced, which are not easily modeled accurately, especially when the operating conditions are transient. The coupling of chemical and biological phenomena to physical models is especially difficult [7,183].

#### 8.2.2. Nonlinear Dynamics and Instability Prediction

MEMS actuators often work in nonlinear regimes where small changes in parameters result in large changes in performance or disastrous failures [272]. The existing models do not usually have a very good ability to minimize the pull-in voltages, resonance shifts, and the dynamic instabilities of the complicated actuator geometries. The memory effects in piezoelectric, magnetic and shape memory actuators are caused by hysteresis effects, making it difficult to design control systems. Dynamically coupled microfluidic systems exhibit chaos and bifurcation effects, which also introduce further modeling problems [163].

#### 8.2.3. Uncertainty Quantification and Reliability Modeling

Uncertainty in the variation in processes and material properties has a very high influence on the performance of the device and is frequently poorly represented in the existing modeling methodologies [273]. It is very difficult to predict long-term reliability with many degradation mechanisms with complex interactions, such as fatigue, creep, and material aging. The creation of effective uncertainty quantification techniques of multi-physics systems is a pressing research requirement. Physics-of-failure models and accelerated testing methods are still to be developed in order to provide accurate lifetime prediction [274].

### 8.3. Data-Guided Optimization and AI-ML in the Actuator Design

This section is not concerned with a hypothetical proposal of a system of smartness, but with how the techniques of data-driven optimization and machine learning have been practically investigated in recent MEMS actuator research and what its current scope, data needs, and limitations are.

#### 8.3.1. Design Optimization with the Help of Machine Learning

Deep learning methods allow for quick actor performance forecasting and optimization of intricate actuator designs that would be computationally inappropriate with conventional methods [275]. GANs and variational autoencoders can be used to investigate new design ideas that are not accessible through human intuition. Adaptive system behavior and the optimization of dynamic control strategies has been demonstrated to be a promising area in reinforcement learning algorithms. Transfer learning techniques enable the transfer of knowledge in one actuator group to quicken the development of new device categories [155,156].

The performance of such methods, however, is highly contingent upon the quality of the simulation or experimental data and most of the reported studies are limited to offline design optimization and not entirely autonomous actuator design.

#### 8.3.2. AI-Powered Control over the Manufacturing Processes and Manufacturing

Deep learning and machine vision allow for defects to be detected in real time and adjustment to the process to occur during fabrication [276]. Equipment optimization and minimization of downtimes in MEMS manufacturing plants are the objectives of predictive maintenance algorithms. Manufacturing data can be used to learn process–structure–property relationships, which can be used to design more robust design-for-manufacturing strategies. Digital twin technology is a technology in which virtual models of fabrication processes are created to optimize and trouble shoot [277].

Currently, AI-aided manufacturing approaches are mostly used at the level of process monitoring and yield improvement, but not at the level of fully self-optimizing MEMS fabrication processes.

#### 8.3.3. Intelligent System Operation and Adaptation

Online learning algorithms make MEMS actuators adjust to the altered operating conditions and performance degradation [158]. Anomaly detection systems are used to detect the imminent failures and deviations in performance before the system fails in a catastrophic manner. Multi-objective optimization algorithms trade off conflicting performance metrics such as speed, precision, power, and reliability. Federated learning models facilitate learning enhancement on both scales across several deployed systems and protect the privacy of data [278].

The majority of reported applications are at a proof-of-concept or laboratory scale, and there are a great deal of issues with robustness, validation, and regulatory acceptance before they can be applied to a working microfluidic system.

### 8.4. Sustainability, Scalability, and Manufacturability Prospects

#### 8.4.1. Sustainable Materials and Green Manufacturing

Increased environmental concerns are related to the creation of lead-free piezoelectric materials, biodegradable polymers, and recyclable device structures [279]. IoT and remote monitoring systems and applications can be self-powered, using energy-harvesting integrated actuators. The green process manufacturing lowers the use of chemicals, energy, and production of hazardous wastes. The concepts of lifecycle assessment and circular economy are being integrated into the MEMS device design [280].

#### 8.4.2. Scalable Technologies in Manufacturing

Roll-to-roll and additive manufacturing allow for the manufacturing of volumes of flexible and disposable microfluidic systems [281]. Heterogeneous integration methods allow for integration of various materials and technologies with improved performance properties. Scalable system integration and upgradability is supported by standardized interfaces and modular architecture. Multi-project wafers and fabs that are shared minimize the cost of development and speed up technology adoption [282].

#### 8.4.3. Advanced Packaging and System Integration

With through silicon vias and wafer level packaging, the density of the integration and performance can be enhanced [137]. MEMS actuators which are monolithically integrated with CMOS control circuitry decrease the system size, cost, and power usage. Flexible hybrid electronics is a combination of the rigid MEMS components and conformal form factors. System-in-package and system-on-chip solutions keep being developed in the direction of greater integration [131].

### 8.5. Future Perspectives of MEMS-Based Microfluidic Systems

The future of MEMS actuator microfluidic applications is the creation of intelligent, adaptive, and multifunctional systems, which are able to smoothly incorporate sensing, actuation and control [8]. The most critical enabling technologies are AI-based design solutions, nanomaterials, heterogeneous integration strategy, and eco-friendly manufacturing. Further growth in innovation and improvement of technology will be stipulated by their emergent use in personalized medicine, environmental monitoring and point-of-care diagnostics.

The production of the next-generation MEMS microfluidic systems involves a tight partnership between materials scientists, device physicists, control engineers and experts of the application domain [283]. Well-integrated cross-disciplinary methods incorporating the knowledge of biology, chemistry and information science will make entirely new capabilities possible. The process of standardization, open-source design software, and common facilities of fabricating will ease the innovation and transfer of technology.

With MEMS actuators being ever-growing in scale, complexity, and intelligence, they will allow for unprecedented fluid control, system control, and manipulation [144]. A combination of learning, adaptive behavior and autonomous operation will turn microfluidic systems into passive instruments of scientific discovery, and technological innovation into active partners in scientific discovery and technology. The next decade is forecasted to see interesting developments that will cement MEMS actuators as an indispensable element in the microfluidic systems of the future.

## 9. Conclusions

This review examined the history and status of MEMS actuators to be applied in microfluidic systems and how novel design methodologies, multiphysics models, and performance optimization can be used. Various diverse actuation mechanisms, including electrostatic and piezoelectric systems, have enhanced integration density, accuracy and functionality by enhancing the material performance of electroactive polymer and magnetic nanocomposites. The multi-material architectures, as well as complex geometries, are made possible by additive manufacturing that evolved to be utilized in the contemporary fabrication techniques, which are founded upon the conventional MEMS. Performance-wise, the existing MEMS actuators for use in microfluidic applications can provide forces on the micro-Newton to sub-milli-Newton scale, and the performance of such actuators can reach displacements in the tens of micrometers to nanometers and response times in the sub-milliseconds to milliseconds time ranges. The operating limits can be effectively useful when fine microfluidic pumping and valving, as well as manipulation of cells, are needed, but this is limited to applications that must operate with a large stroke or produce large forces. The idea that the requirement for design multiphysics would give the true modeling of actuators, and predicting their behavior through high-fidelity simulations and multi-objective optimization models, is one of the themes that was found in this review. Positive indicators of multifunctional architectures that are concurrent in sensing and actuation, AI-assisted design tools, reliability, and sustainability also exist. However, there are a number of research gaps that need to be addressed, such as small actuator stroke (usually less than 100 um), small force output in compact MEMS devices (less than 1 mN), long-term reliability issues, and standardized benchmarking measures that can be used to compare and contrast different actuator technologies fairly. In the future, the momentum of progress is likely to be based on the multidisciplinary methods to incorporate the materials innovation, multiphysics modeling, data-driven design tools and the establishment of standardized performance metrics to aim at a higher level of reproducibility, manufacturability, and scalability. Further evolution in the future will be formulated on the basis of a multidisciplinary approach and the creation of standardized benchmarks to enhance the reproducibility and commercialization, aiming at attempting to transform laboratory innovations into scalable systems with clinical feasibility.

## Figures and Tables

**Table 1:** Comparison of piezoelectric actuation platforms of MEMS-based microfluidic systems in order of magnitude [42,45,49,50,51].

Platform	Typical Drive Voltage	Strain/Stroke	Bandwidth
Thin-film piezo MEMS (AlN/AlScN/thin-film PZT)	5–50 V	~0.1–10 µm (direct)	kHz–MHz
Bulk/stack PZT actuators	50–200 V	~1–100 µm (direct)	kHz
Mechanically amplified piezo actuators	10–200 V	~0.1–1 mm (effective)	Hz–kHz

**Table 2:** Benchmarking of electromagnetic actuation for MEMS-based microfluidic systems [55,62,63].

Parameter	Order-of-Magnitude Value	Notes
Drive current/voltage	10–100 mA/0.5–5 V	Current-driven; higher current increases force but raises Joule heating
Force output	10 µN–1 mN	Suitable for membrane pumping and valving
Displacement (stroke)	1–100 µm	Larger strokes require membranes or mechanical amplification
Bandwidth	10 Hz–1 kHz	Limited by mechanical mass and fluid damping
Power consumption	1–100 mW	Dominated by resistive (Joule) losses

**Table 3:** Benchmarking of key performance, scalability, and fabrication metrics for MEMS actuators in microfluidic systems.

Actuator Type	Force	Displacement	Power	Scalability	Fabrication	Advantages	Limitations	References
Electrostatic	µN–100 µN	0.1–10 µm	µW–mW	High	Low–Medium	Fast, low power, CMOS-compatible	Pull-in, liquid reliability	[24,26]
Electrothermal	10 µN–1 mN	1–50 µm	10–100 mW	Medium	Low	High force, simple design	Heating, low efficiency	[31,32]
Piezoelectric	10 µN–1 mN	0.1–100 µm	~mW	Medium	High	Fast, precise, low heating	Cost, thin-film variability	[41,45]
Electromagnetic	10 µN–1 mN	1–100 µm	1–100 mW	Low	High	Linear force, high stroke	Bulky, power hungry	[55,64]
EWOD	nN–µN	Droplet motion	µW–mW	High	Medium	No moving parts, digital control	Fouling, voltage limits	[70,71,74]
Pneumatic	mN+	10–100 µm	External	Very High	Medium	Gentle, scalable	External hardware	[74,76,79]

**Table 4:** Comparison of structural materials for MEMS microfluidic actuators.

Material Category	Young’s Modulus (GPa)	Thermal Expansion (ppm/°C)	Biocompatibility	Applications	Limitations	References
Single Crystal Si	160–190	2.6	Good	High-precision actuators, pumps	Brittle, limited transparency	[101,103]
SOI Wafers	160–190	2.6	Good	Complex MEMS structures	Higher cost, processing complexity	[102]
Borosilicate Glass	60–70	3.3	Excellent	Optical detection, chemical reactors	Low fracture toughness	[2,104]
PDMS	0.00036–0.0025	310	Excellent	Rapid prototyping, pneumatic valves	Swelling in organic solvents	[107,111]
PMMA	2–3	50–90	Good	Disposable chips, optical devices	Limited chemical resistance	[108,112]
SU-8	~4.0	52 ± 2	Good	High-aspect-ratio structures	Stress-induced cracking	[113]
PEEK	3–4	40–60	Excellent	Chemical reactors, implantables	High processing temperature	[107]
Paper	1-10	-	Good	Point-of-care diagnostics	Limited mechanical strength	[108]

**Table 5:** Comparison of functional materials for MEMS actuators.

Material Type	Properties	Actuation Mechanism	Advantages	Limitations	References
PZT	d33 = 400–650 pC/N	Piezoelectric	High force, fast response	Contains lead, brittle	[114,115,123]
AlN	d33 = 5–6 pC/N	Piezoelectric	CMOS compatible, lead-free	Lower piezoelectric coefficient	[42,116,124]
AlScN	d33 = 20–30 pC/N	Piezoelectric	Enhanced performance, compatible	Higher cost, process development	[42,43]
IPMC	Strain > 5%	Electrochemical	Large deformation, low voltage	Requires hydration, slow response	[118,119]
Permalloy	μ_r_ > 50,000	Magnetic	High permeability, low loss	Requires external magnetic field	[55]
NdFeB	BH_max_ > 40 MGOe	Magnetic	Strong permanent magnet	Difficult to pattern, corrosion	[55,57]
ITO	ρ = 10–100 Ω/sq	Conductive	Transparent, stable	Brittle, limited flexibility	[81]
PEDOT	ρ = 0.1–100 Ω·cm	Conductive	Flexible, solution processable	Stability issues in humidity	[121]
PVDF (Polyvinylidene fluoride)	d33 ≈ 20–30 pC/N	Piezoelectric	Flexible, lightweight, biocompatible	Lower force than PZT	[123,125]

**Table 6:** Comparison of fabrication methods for MEMS microfluidic actuators.

Fabrication Method	Minimum Feature Size	Aspect Ratio	Material Compatibility	Throughput	Cost	References
Bulk Micromachining	1–2 μm	10–50	Si, Glass	Medium	High	[2,11,128]
Surface Micromachining	0.5–1 μm	5–20	PolySi, Metals	Low	High	[2,127,131]
DRIE	0.1–1 μm	10–100	Si, Some metals	Medium	High	[2,11,104]
Soft Lithography	0.1–1 μm	1–5	PDMS, Polymers	High	Low	[75,107,113]
Hot Embossing	0.1–1 μm	1–10	Thermoplastics	High	Medium	[108,113,129]
Injection Molding	1–10 μm	1–5	Thermoplastics	Very High	Low (mass production)	[108,113,129,132]
Two-Photon Polymerization	0.1–0.5 μm	1–20	Photopolymers	Very Low	High	[130]
Micro-SLA	1–10 μm	1–5	Photopolymers	Medium	Medium	[113,130]

**Table 7:** Comparison of integration and packaging methods.

Packaging Method	Bond Strength	Temperature Resistance	Chemical Resistance	Reusability	Applications	References
Anodic Bonding	Very High	>400 °C	Excellent	No	Silicon–glass permanent seals	[106]
Thermal Bonding	High	>800 °C	Excellent	No	High-temperature applications	[112,113]
Adhesive Bonding	Medium	<150 °C	Good to Poor	No	Multi-material bonding	[113,132]
Plasma Bonding	Medium–High	<100 °C	Fair	Limited	PDMS devices, prototyping	[106,107]
Mechanical Clamping	Low	—	Excellent	Yes	Testing, reconfigurable systems	[2]
Solvent Bonding	Medium	<80 °C	Poor	No	Thermoplastic bonding	[112]
UV Curing	Medium	<120 °C	Good	No	Rapid assembly, low temperature	[107]
Laser Bonding	High	Localized heating	Excellent	No	Glass/polymer microfluidics	[132,137]
Frit Bonding	Very High	>400 °C	Excellent	No	MEMS and microfluidic devices	[137]

**Table 8:** Representative multiphysics coupling models and modeling limitations of MEMS microfluidic actuators [165,167,168,169,170,171,172,173,174,175,176,177].

Coupling Type	Governing Equations	Key Phenomena Captured	Tools/Methods	Modeling Limitations/Gaps
Electromechanical	Poisson’s and Elasticity Equation	Pull-in instability, frequency shift	FEM (COMSOL, ANSYS)	Dielectric charging drift; sensitivity to gap/residual stress; viscous damping in liquid
Thermo-Fluidic	Navier–Stokes + Heat Equation	Bubble growth, thermocapillary flow	CFD, ALE mesh	Nucleation/phase-change uncertainty; high computational cost; parameter sensitivity
Piezo–Electromechanical	Gauss’s + Constitutive Equation	FSI, hysteresis	Coupled FEM/CFD	Hysteresis/nonlinear constitutive behavior; thin-film variability; calibration need

**Table 9:** Optimization of the MEMS microfluidic actuator, using performance measures and modeling [31,34,41,165,174,177,217,224,225,226,227,228].

Metric	Definition/Importance	Dominant Design Factors	Typical Modeling Approach
Actuation Force	Maximum back-pressure or sealing pressure a pump/valve can overcome	Piezoelectric material constants (PZT-5H, AlN), diaphragm thickness, electrothermal beam geometry	FEM structural solver, electromechanical coupling
Displacement/Stroke	Volume displaced per actuation cycle; affects throughput	Membrane stiffness, flexure hinges, corrugated/bimorph design, chamber geometry	FSI + nonlinear FEM membrane deformation
Response Time	Switching or settling time for actuator motion	Resonant frequency, damping, thermal RC constant, squeeze-film losses	Harmonic FEM, thermal–mechanical transient CFD
Energy Efficiency	Flow-rate-per-watt or force-per-watt delivered	Diffuser geometry, driver electronics, thermal loss, material conversion efficiency	FEM-CFD power analysis, material coupling

## Data Availability

Data sharing is not applicable to this article as no datasets were generated or analyzed during the current study.
